# Mutational spectrum and risk stratification of intermediate-risk acute myeloid leukemia patients based on next-generation sequencing

**DOI:** 10.18632/oncotarget.7028

**Published:** 2016-01-27

**Authors:** Bianhong Wang, Yangyang Liu, Guangyuan Hou, Lili Wang, Na Lv, Yuanyuan Xu, Yihan Xu, Xiuli Wang, Zhaoling Xuan, Yu Jing, Honghua Li, Xiangshu Jin, Ailing Deng, Li Wang, Xiaoning Gao, Liping Dou, Junbin Liang, Chongjian Chen, Yonghui Li, Li Yu

**Affiliations:** ^1^ Medical Center, Tsinghua University, Beijing 100084, China; ^2^ Department of Hematology, Chinese PLA General Hospital, Beijing 100853, China; ^3^ Research and Development Department, Annoroad Gene Technology Co. Ltd, Beijing 100176, China

**Keywords:** intermediate-risk acute myeloid leukemia, next generation sequencing, mutational screening and analysis, risk stratification

## Abstract

Intermediate-risk acute myeloid leukemia (IR-AML), which accounts for a substantial number of AML cases, is highly heterogeneous. Although several mutations have been identified, the heterogeneity of AML is uncertain because novel mutations have yet to be discovered. Here we applied next generation sequencing (NGS) platform to screen mutational hotspots in 410 genes relevant to hematological malignancy. IR-AML samples (N=95) were sequenced by Illumina Hiseq and mutations in 101 genes were identified. Only seven genes (*CEBPA, NPM1, DNMT3A, FLT3-ITD, NRAS, IDH2* and *WT1*) were mutated in more than 10% of patients. Genetic interaction analysis identified several cooperative and exclusive patterns of overlapping mutations. Mutational analysis indicated some correlation between genotype and phenotype. *FLT3-ITD* mutations were identified as independent factors of poor prognosis, while CEBPA mutations were independent favorable factors. Co-occurrence of *FLT3-ITD, NPM1* and *DNMT3A* mutations was identified with associated with specific clinical AML features and poor outcomes. Furthermore, by integrating multiple mutations in the survival analysis, 95 IR-AML patients could be stratified into three distinct risk groups allowing reductions in IR-AML by one-third. Our study offers deep insights into the molecular pathogenesis and biology of AML and indicated that the prognosis of IR-AML could be further stratified by different mutation combinations which may direct future treatment intervention.

## INTRODUCTION

Acute myeloid leukemia (AML) is characterized by the accumulation of somatically acquired genetic changes in hematopoietic progenitor cells, including gene mutations, copy number alterations and chromosomal translocation [1, 2]. Epigenetic aberrations, including methylation and genomic miRNA deregulation, also play an important role in AML classification and risk stratification. Traditionally, chromosome karyotype has been accepted to be the most important prognostic parameter in AML. However, nearly half of AML cases have a normal karyotype at diagnosis and are categorized as the intermediate-risk (IR) although they have significant clinical heterogeneity [3]. It is believed, therefore, that genetic mutational analysis may help these patients. Advances in identification of prognostic genetic alterations have facilitated greater detailed risk stratification [4, 5]. Previous studies have focused on several selected genes, for example, *FLT3-ITD, NPM1* and *CEBPA* mutations have been incorporated in the European LeukemiaNet (ELN) risk classification system [6].A recent study by Hou and colleagues [7] reported that IR-AML patients could be re-stratified into three distinct prognostic groups according to the mutation status of *FLT3-ITD, NPM1, CEBPA, IDH2, WT1, ASXL1, RUNX1* and *DNMT3A.*

Because tumor cells harbor hundreds of mutated genes and multiple mutations that often occur concomitantly, mutational screening for a panel of genes is biologically meaningful. Traditional sequencing platforms require large amounts of DNA to assess only one gene at a time, and it is labor-intensive and time-consuming, as well as logistically difficult for data integration in real time. Recently, next generation sequencing (NGS) has been shown to have great advantage and potential [8, 9] because of its massively parallel sequencing ability and high throughput multiplexing capacity, making simultaneously parallel and targeted sequencing of all genes of interest feasible, suggesting its value in routine clinical practice [10, 11]. One study recently conducted by the Cancer Genome Atlas Research Network analyzed the genomes of 200 adult AML patients by NGS, and mutations were classified into 9 categories. Nearly all AML samples had at least one mutation of these categories, and a complex interplay of genetic alterations was identified, partly explaining the genetic aberrations in AML. Thus, this approach will significantly impact future disease classification systems [12], but currently no such research is specifically directed toward IR-AML.

In this study, we applied a NGS platform to screen for mutations in 410 genes relevant to hematological malignancy and showed the value of this approach. Furthermore, we performed single and comprehensive mutational analyses associated with clinical features and propose new risk stratification model for IR-AML and its distinct molecular subgroups that will benefit from personalized therapy.

## RESULTS

### Clinical characteristics of the patients and panel genes

Clinical characteristics of the 95 patients are summarized in Table 1. Among these patients, fifty-five were males and forty were females. The median age was 45 years, ranging from 12 to 88 years and there were ninety-two adults and three children (≤15years). The median follow-up was 22.3 months (interquartile range, 7.5–38 m). Among our 95 IR-AML patients, excluding 10 patients who did not receive any chemotherapy or were only treated with low-dose cytosine arabinoside because of old age and/or poor performance status, sixty-five patients received conventional induction chemotherapy with one of the anthracyclines (idarubicin or doxorubicin) or mitoxantrone for 3 days and cytarabine for 7 days, another twenty patients received DCAG (decitabine 10 mg/m^2^ d1-5, aclarubicin 20mg d1,3,5, cytarabine 10mg/m^2^ q12h d1-5, G-CSF 300μg/day) regimen. After achieving complete remission, twenty-five patients received consolidation chemotherapy with conventional dose of cytarabine and anthracycline or mitoxantrone or with middle-/high-dose cytarabine and forty patients received hematopoietic stem cell transplantation for consolidation chemotherapy. Among them, 75 patients receiving more than 4 cycles of chemotherapy were used for survival analysis.

**Table 1:** Clinical and pathologic characteristics of 95 IR-AML patients

Characteristic	Median(range) or Number
**Age at study entry (years)**	45 (12–88)
**Sex, no. (%)**	
Male	55/95
Female	40/95
**White-cell count at diagnosis**	
WBC-G/L	21.33 (0.87–405.13)
Bone marrow blast at diagnosis, %	64.4 (20–94.4)
**AML FAB subtype-no. (%)**	
AML with minimal maturation: M0	0
AML without maturation: M1	4/95
AML with maturation: M2	23/95
Acute myelomonocytic leukemia: M4	28/95
Acute monoblastic or monocytic leukemia: M5	31/95
Acute erythroid leukemia: M6	5/95
Unclassified	4/95
**Immunophenotype**	
CD13	73/95
CD33	86/95
CD34	68/95
CD117	77/95
MPO	77/95
**Cytogenetics**	
Abnormal karyotype	20/95
Normal karyotype	75/95
**Induction therapy**	
IA/DA/MA	65/95
DCAG	20/95
Others	10/95
**Response evaluation**	
Achieving CR	65/95
Non-remission	22/95
Unevaluated	8/95
**Consolidation therapy after CR**	
Chemotherapy	25/65
HSCT	40/65

Abbreviation: WBC, white blood cell count. FAB, French American British. CR, complete remission. IA, idarubicin+ cytarabine. DA, daunorubicin+ cytarabine. MA, mitoxantrone+cytarabine. DCAG, decitabine+aclarubicin+cytarabine+ G-CSF. HSCT, hematopoietic stem cell transplantation.

The sequencing panel targets ~1.3 Mb of genomic content, consisting of the entire coding sequence of 410 genes relevant to the pathogenesis of hematologic malignancies. For this panel, 233 genes were originated from the COSMIC cancer database which contained somatic mutations in human blood cancer, and 23 genes originated from the Leukemia Gene Database (LeGenD). In addition, 154 genes were collected based on recent scientific and clinical literature (Supplementary Table S1).

### Sensitivity of mutation detection

**Figure 1:**
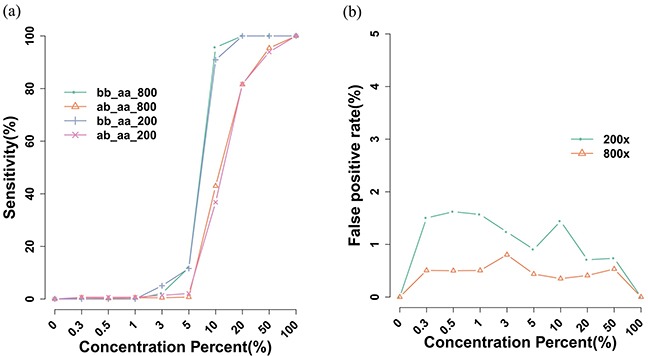
Sensitivity and false-positive mutation detection rates **a.** In Kasumi-1 and K562 cell lines, mutation sensitivity can be calculated and more than 80% of unique mutations within Kasumi-1 can be detected. **b.** The false-positive rate revealed that more than 98.5% mutations were consistent with Kasumi-1 cell line data.

To create a 0–100% concentration gradient of tumor mutations in the Kasumi-1 cell line, Kasumi-1 cells were diluted with various ratios of K562 cells. Sequencing results from two different depths indicated at least 90% of the homozygous mutations in the cell line were correctly detected in a 10% solution of K562 cells and that 80% of the heterozygous mutations were identified in the 20% K562 cell solution. In contrast, false-positives were detected at 0 and 100% K562 cell solutions. Using the cell line alone, false-positives were less than 1% (Figure 1). In all IR-AML samples, tumor cells comprised more than 20% of the sample. Thus, detecting mutations is efficient when covering sequences above 200x. We counted allelic frequencies of unique SNPs in Kasumi-1 cells at each dilution and there was a concordance with each dilution which reflected the tumor concentration (Supplementary Figure S1).

### Frequencies of genetic alterations

In total, 8,766 single-nucleotide variants (SNVs) and 839 small insertion and deletions were detected in 95 IR-AML patients for the target region in the absence of matched control samples. We focused on the mutations that change with respect to exon amino acid coding regions, consensus splice-site regions, and RNA genes. A series of steps were used to remove germline and harmless mutations (Supplementary Methods). After filtering, an average of 3.6 mutations per sample were remained and 101 of 410 genes analyzed were identified (Supplementary Table S2). Each mutation position and gene type of some genes are shown in Supplementary Figure S2. Due to the limited amount of normal control samples, we did not confirm that all identified mutations were somatic mutations, so some may be rare SNPs. *CEBPA* and *NPM1* were the two most commonly identified mutations (28.4% and 21.1% respectively), followed by *DNMT3A* (16.8%), *FLT3-ITD* (14.7%), *NRAS* (12.6%), *IDH2* (11.6%) and *WT1* (10.5%) mutations. Mutations of some genes like *DNMT3A*, *IDH2*, *NRAS*, *TET2* and *WT1* were shown in Supplementary Table S3 and these results were all validated by Sanger sequencing.

**Figure 2:**
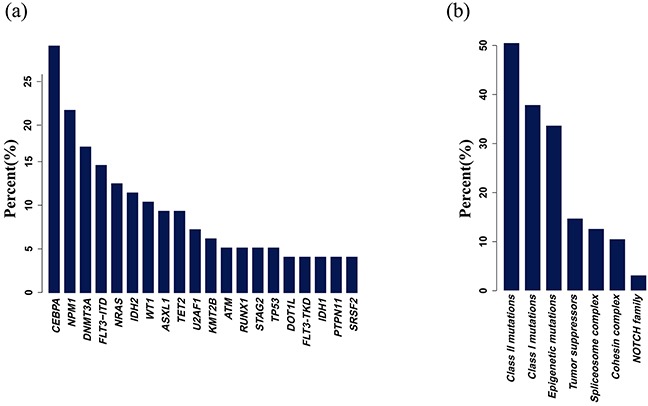
Mutation frequencies **a.** Mutation frequencies that occurred in more than four samples. Mutations were identified in 101 of 410 genes analyzed. Only seven genes (*CEBPA, NPM1, DNMT3A, FLT3-ITD, NRAS, IDH2* and *WT1*) were mutated in more than 10% of patients. **b.** Mutation frequencies according to functional classification. Mutations in class I and II and epigenetic modifiers were frequently identified.

Conceptually, genetic alterations identified in AML have been grouped into class I mutations that promote growth and survival by activating intracellular signals, and class II mutations that block differentiation, impair subsequent apoptosis, and/or confer an advantage in self-renewal by altered transcription factors [13]. Presently, other family gene mutations have been classified according to their provisional gene function, such as epigenetic modifiers, tumor suppressors, the cohesin complex, and spliceosome genes [9, 12, 14]. In this study, class II mutations (*NPM1*, *CEBPA*, *RUNX1*, *GATA2*, *ETV6*, *etc*) were the mostly frequently identified (48/95, 50.53%), followed by class I mutations (*FLT3*, *KIT*, *NRAS*, *KRAS*, *PTPN11*, *etc*; 36/95, 37.89%), epigenetic modification mutations (*DNMT3A*, *TET2*, *IDH1/2*, *ASXL1*, *EZH2*, *DOT1L*, *etc*; 32/95, 33.68%), and tumor suppressor mutations (*WT1*, *PHF6* and *TP53*) (14/95, 14.74%). Also, spliceosome genes (*U2AF1*, *SRSF2* and *SF3B1/2*), cohesion complex genes (*STAG2*, *RAD21*, *SMC1A* and *SMC3*) and NOTCH family mutations were identified in 12 (12.63%), 10 (10.53%) and 3 (3.16%) patients, respectively (Figure 2).

### Interaction of genetic alterations

**Figure 3:**
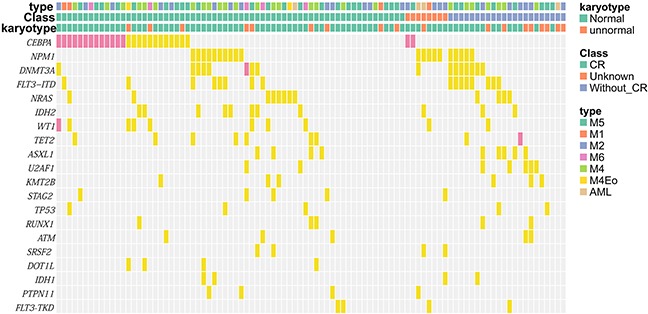
Mutation profile according to clinical features Mutation profile according to clinical features (FAB category, clinical efficacy and karyotype). Significant mutations identified in AML patients are shown. Some mutations co-occurred or were exclusive and some were associated with specific clinical features. Yellow boxes indicate single mutations and pink boxes indicate double mutations.

Mutation profiles and several patterns of overlapping mutations were identified in our study. (Figure 3, Supplementary Figure S3). Significantly overlapped mutations were observed among *FLT3-ITD*, *NPM1* and *DNMT3A* mutations; *NRAS and WT1* mutations. In contrast, mutually exclusive mutations were observed between *TET2* and *IDH1/2* mutations; *WT1* and *TET2* mutations; *NPM1* and *CEBPA* mutations; and between *STAG2* and *SMC3* mutations. Overall, we observed relationships of strong co-occurrences between class I and II and epigenetic modifying gene mutations, which also often coexisted with other family gene mutations such as the cohesion complex, *NOTCH* family and spliceosome mutations [8]. In contrast, mutations of the same gene classification were always mutually exclusive.

### Clinical features of single mutational analysis

Several associations between mutations and clinical features were observed. *DNMT3A* mutations occurred more frequently in older patients (>50 years-of-age, *p*<0.05) and *NPM1* mutations were more frequently identified in patients older than 40 years-of-age (*p*<0.05). In addition, several mutations were identified to be associated with peripheral white leukocyte and bone marrow blast counts at diagnosis. *FLT3-ITD* was associated with high peripheral leukocyte counts and high numbers of bone marrow blast cells (*p*<0.05). In contrast, *IDH2* mutations were associated with a lower leukocyte count (*p*<0.05) and some mutations were found to be associated with specific immunophenotype. Compared with the non-mutation group, *NPM1* mutations were associated with lower CD34- and HLA-DR-positive rates (*p*<0.05) but with higher CD33-positive rates (*p*<0.05). *DNMT3A* mutations were associated with fewer CD34-, CD33- and CD117-positive rates (*p*<0.05). *CEBPA* mutations were associated with greater CD34- and CD7-positive rates (*p*<0.05) and *IDH2* mutations were associated with fewer HLA-DR-positive rate (*p*<0.05) (Supplementary Table S4).

**Figure 4:**
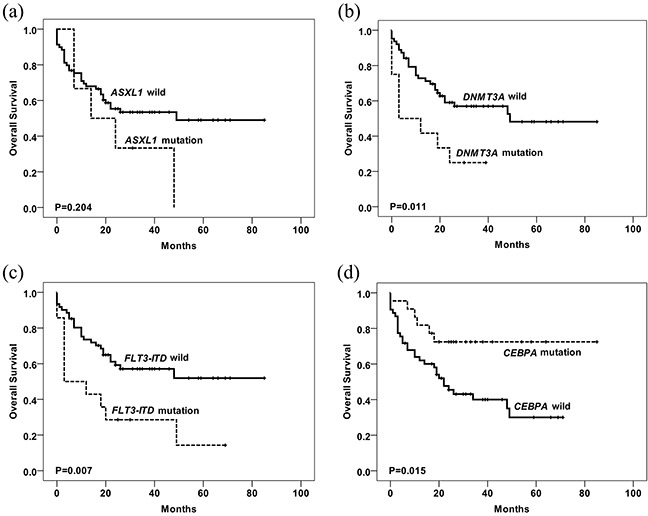
Kaplan-Meier curves of OS according to the mutations are shown Overall survival stratified by mutational status. P value was estimated by the log-rank test. The mutated number of *ASXL1* **(a)**, *DNMT3A* **(b)**, *FLT3-ITD* **(c)** and *CEBPA* **(d)** for survival analysis was 6, 12, 14 and 22, respectively. Patients with *ASXL1*, *DNMT3A* or *FLT3-ITD* mutations have worse survival than wild type groups, while patients with *CEBPA* mutations have better OS than those without mutations.

**Figure 5:**
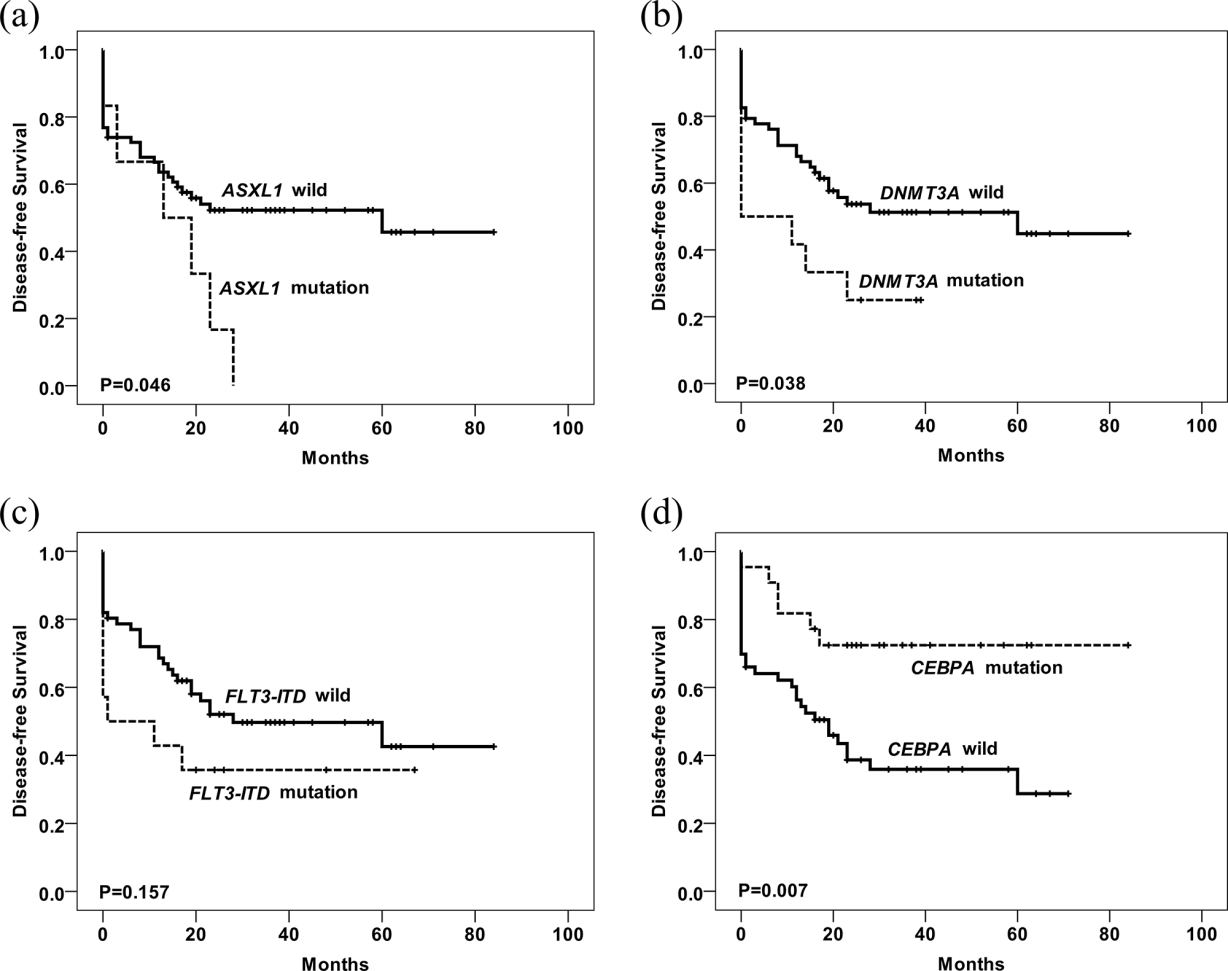
Kaplan-Meier curves of DFS according to the mutations are shown Disease-free survival stratified by mutational status. P value was estimated by the log-rank test. The mutated number of *ASXL1* **(a)**, *DNMT3A* **(b)**, *FLT3-ITD* **(c)** and *CEBPA* **(d)** for survival analysis was 6, 12, 14 and 22, respectively. Patients with *ASXL1*, *DNMT3A* or *FLT3-ITD* mutations have worse survival than wild type group, while patients with *CEBPA* mutations have better DFS than those without mutations.

We also analyzed the association between mutations and CR. Using Pearson's χ^2^ test, *CEBPA* mutations were identified as a favorable factor for achieving CR, whereas *ASXL1*, *FLT3-ITD* and *DNMT3A* mutations were unfavorable factors (Table 2). Multivariable logistic regression analysis showed that only *FLT3-ITD* and *ASXL1* mutations were identified as unfavorable factors for achieving CR. We then analyzed the prognostic value of single-gene mutations. Using univariate analysis, *FLT3-ITD, ASXL1* and *DNMT3A* mutations were identified as unfavorable prognostic factors for overall survival (OS) and disease-free survival (DFS), and *CEBPA* mutations were favorable factors for OS and DFS, while some other genes were not identified as prognosis factors (Figure 4, Figure 5, Table 3, Supplementary Figure S4). Multivariate COX regression analysis with stepwise selection showed that *FLT3-ITD* (HR: 3.271, 95% CI: 1.541-6.944, *P*=0.002) mutations were independent factors of poor prognosis, and *CEBPA* (HR: 0.407, 95% CI: 0.168-0.985, *P*=0.046) mutations were independent favorable factors.

**Table 2:** Gene mutations affecting CR

Mutations	*CR rate (%)*		*P*-value
*Pearson's* χ*2 test*	*Positive*	*Negative*	
*ASXL1*	4/9(44.4%)	61/78(78.2%)	0.042
*DNMT3A*	8/15(53.3%)	57/72(79.2%)	0.036
*FLT3-ITD*	7/14(50.0%)	58/73(79.5%)	0.020
*CEBPA* *NPM1*	25/25(100%) 10/15(66.7%)	40/62(64.5%) 55/72(76.4%)	0.001 0.434
**Mutations**	***OR (95% CI)***		***P*-value**
*Multivariate analysis*			
*ASXL1*	0.148(0.034–0.649)		0.011
*FLT3-ITD*	0.185(0.053–0.644)		0.008

Abbreviations: CR, complete remission; OR, odds ratio; CI, confidence interval. By Pearson's χ^2^ test, *CEBPA* mutations were identified as favorable factors for achieving CR, while *ASXL1*, *DNMT3A*, *FLT3-ITD* mutations were as unfavorable factors. Multivariable logistic regression analysis showed that only *FLT3-ITD* and *ASXL1* mutations were identified as unfavorable factors for achieving CR.

**Table 3:** Univariate and Multivariate Analysis for DFS and OS

	Univariated analysis		Multivariate analysis	
	*P*-value	Log rank χ^2^ *test*	*P*-value	HR (95% CI)
**OS**				
Age>*50y*	0.001	11.574	>0.1	
*ASXL1*	0.204	1.617	>0.1	
*DNMT3A*	0.011	6.399	>0.1	
*FLT3-ITD*	0.007	7.396	0.002	3.271 (1.541–6.944)
*CEBPA*	0.015	5.939	0.046	0.407 (0.168–0.985)
HSCT	<0.001	27.315	<0.001	0.151 (0.068–0.337)
WBC count>30G/L	0.066	3.368	>0.1	
**DFS**				
Age>*50y*	0.001	11.613	>0.1	
*ASXL1*	0.046	3.965	>0.1	
*DNMT3A*	0.038	4.293	>0.1	
*FLT3-ITD*	0.157	1.999	>0.1	
*CEBPA*	0.007	7.245	0.022	0.360 (0.150–0.865)
HSCT	<0.001	28.360	<0.001	0.191 (0.090–0.406)
WBC count>30G/L	0.404	0.697	>0.1	

Abbreviations: P of the analysis is the p value of the Log rank test. HR is the value of the hazard ratio. 95% CI is the 95% confident interval of the hazard ratio.WBC, white blood cell count. HSCT, hematopoietic stem cell transplantation.

### Clinical features of comprehensive analysis of multiple mutations

In our study, the most notably significant co-occurrence was identified among *FLT3-ITD*, *NPM1* and *DNMT3A* mutations, which occurred in five patients (Table 4). *NPM1^mut^*/FLT3-ITD*^mut^*/DNMT3A*^mut^* patients were older (median age, 61 years), mostly women (4/5, 80%) and had a heavy disease burden with greater leukocyte counts (*p*<0.05, median, 132.39×10^9^/L) than *NPM1^wt^*/*FLT3-ITD^mut^*/*DNMT3A*^mut^, *NPM1^wt^*/*FLT3-ITD^neg^*/*DNMT3A^mut^* or *NMP1^mut^*/*FLT3-ITD^neg^*/*DNMT3A^mut^* groups (Supplementary Table S5). Morphologically, *NPM1^mut^*/FLT3-ITD*^mut^*/ DNMT3A*^mut^* AML was closely associated with myelomonocytic blast morphology, corresponding to M4 and M5 categories in the French-American-British (FAB) classification [15]. Among these five cases, three (60%) were M4 and two (40%) were M5. In terms of immunophenotype, all cases were positive for CD33 and CD13 and negative for surface and cytoplasmic CD3 expression. Myeloperoxidase expression was measured in 3/5 (60%) cases and blasts were positive for CD34 or CD56 in 2/5 (40%) cases. In accordance with myelomonocytic morphology, all cases expressed monocyte-associated CD64 antigen, of which two cases expressed additional monocyte-associated CD14 antigen. Additionally, no case was co-expressed with the B-cell marker CD19. Regarding prognosis, all five patients were non-remission after induction chemotherapy, and all died within six months (average survival time<3 m; range: 0.1-3.5 m).

**Table 4:** Clinical features of 5 patients with concurrent *FLT3-ITD*, *NPM1* and *DNMT3A* mutations

Clinical features	No. of Samples				
	D-2081	D-2959	D-2978	D-3128	D-3009
Age (y)	73	61	43	56	66
Sex	male	female	female	female	female
Diagnosis	M4	M5	M4	M4	M5
WBC at diagnosis (×10^9^/L)	129.21	170	332.77	132.39	70
BM percentage (%)	80.40	68.40	88.40	48	85
Immunophenotype					
CD33	+	+	+	+	+
CD13	+	+	+	+	+
MPO	-	-	+	+	+
CD3	-	-	-	-	-
CD34	-	-	+	+	-
CD56	+	+	-	-	-
CD64	-	+	+	+	+
CD14	-	+	+	-	-
CD19	-	-	-	-	-
*DNMT3A*^mut^	R882H	R882C	R882P	R882H	A910V
*NPM1* insertion type	TCTG	TCTG	TCTG	TCTG	TGCA
Response evaluation	NR	NR	NR	NR	NR
Survival time	3.4 m	3 m	2 d	1 w	3.4 m

In addition, another five patients had *NPM1*^mut^/*IDH 1/2*^mut^ without *FLT3-ITD* (See Table 5). In these patients, blasts were positive for CD33 and myeloperoxidase and negative for surface and cytoplasmic CD3 expression. CD34 and CD56 expression occurred in one patient. All five patients had low leukocyte counts (range: 1.35-49.4×10^9^/L). The four patients available for analysis all achieved CR.

**Table 5:** Clinical features of 5 patients with concurrent *NPM1* and *IDH1/2* without *FLT3-ITD*

Clinical features	No. of Samples				
	D-2842	D-2848	D-2857	D-2862	D-2954
Age (y)	53	36	50	47	54
Sex	female	female	male	female	male
Diagnosis	M5	M2	M4	M4	AML
Diagnosis time	2011.8.15	2012.10.8	2012.7.4	2013.1.17	2010.9.20
WBC at diagnosis(×10^9^/L)	18.01	4.39	14.07	1.35	49.4
BM percentage (%)	84.2	64.4	53.6	92.8	84
Immunophenotype					
CD33	+	+	+	+	+
CD13	+	+	+	-	+
MPO	+	+	+	+	+
CD3	-	-	-	-	-
CD34	+	-	-	-	+
CD56	+	-	+	-	-
Response evaluation	CR	CR	CR	CR	Unevaluated

For overall prognosis, we evaluated a recently reported risk stratification system based on genetic status proposed by Patel's group [4]. According to their system, our 95 IR-AML patients were clearly stratified into 3 distinct prognostic subgroups: a favorable group that accounts for 5.3%, an intermediate group, 70.5%, and a new emerging 24.2% unfavorable group. We then analyzed combinations of different mutations with respect to OS and these data agreed with previous reports (Supplementary Figure S5).

## DISCUSSION

AML is a genetically heterogeneous disease resulting from complex interactions among different oncogenic pathways in leukemogenesis, so integrated mutational profiling via mutational analysis is highly valuable for thorough evaluation [6, 12]. In addition, one gene often has multiple mutation events and traditional sequencing platforms are challenging. NGS technology provides advantages of parallel sequencing and high throughput multiplexing ability, facilitating routine and simultaneous parallel and targeted sequencing of all genes. In our study, cell dilution data indicated that our current pipeline can detect genomic aberrations when at least 10% tumor content is present, and it is sufficient for any *de novo* non-M3 AML patients. The tumor allelic frequency can reflect the concentration of tumor correctly, even as dilute as 0.3%.

Using a second detection method, for each cell line dilution, we observed that at 10%, 100% homozygous and 93% heterozygous mutations were correctly detected in 800x coverage data. With more coverage, detection sensitivity can be much lower than 10%. The lowest detection sensitivity we achieved was 1.5% with a 50% detection rate (Supplementary Figure S6). Other methods may offer detection as low as 0.02% but detection rates are not reported [16]. Thus, our approaches might have potential for detecting minimal residual disease.

In this study, we comprehensively analyzed mutations of 410 genes using a NGS platform and identified 9,605 mutations in 95 IR-AML samples. Consistent with previous reports [4, 8, 17, 18]], only seven genes (*CEBPA*, *NPM1*, *DNMT3A*, *FLT3-ITD*, *NRAS*, *IDH2* and *WT1*) were mutated in more than 10% of study patients but *CEBPA* mutations were more frequent [19]. This finding may be due to patient selection as all patients belong to the intermediate risk group and might be related with racial factors. Likewise, the *CEBPA* mutation rate in CN-AML that reported in Taiwan was also higher, that is, about 35% [20]. Moreover, we analyzed another 26 newly diagnosed AML who own bone marrow and paired control saliva samples to verify the reliability of our method. The result showed that the mutation frequency of genes by two methods (with or without matched control samples) was almost consistent (Supplementary Figure S7), especially for those clinically relevant and important genes with recognized mutation hotspots [21–25], suggesting that the variants of our 95 IR-AML cases represent somatic mutations. In our study, *DNMT3A* was identified as being frequently overlapped with *FLT3-ITD* and *NPM1* mutations. In contrast, *TET2* and *IDH1/2* mutations, *WT1* and *TET2* mutations, *NPM1* and *CEBPA* mutations, and cohesion complex genes (*STAG2* and *SMC3*) were found to be mutually exclusive, and this is consistent with biological evidence that these mutations are functionally involved in a shared mechanism of hematopoietic transformation [26, 27]. Such cooperative and exclusive mutational patterns may indicate a definite role in the pathogenesis of AML, but additional studies are required for further validation.

We observed correlation between mutations and immunophenotype. For example, *NPM1^mut^* patients had higher CD33-positive rates and fewer CD34- and HLA-DR-positive rates, whereas *CEBPA* mutations were associated with high expression of CD34 and CD7. This is the first report to document this observation [28, 29]. Consistent with other studies [30], all four patients with *NPM1^mut^*/FLT3-ITD*^mut^*/DNMT3A*^mut^* were positive for CD33 and CD13, and negative for surface and cytoplasmic CD3 expression. Additionally, another study [31] indicated that patients with *NPM1^mut^* /*FLT3^wt^* were always CD34- and CD56-; while those with *NPM1^wt^* /*FLT3-ITD^mut^* were CD34+ and TdT+. However, we did not observe this in our study. Our results showed some specific correlation between mutation and phenotype, but deducing the phenotypic features from specific somatic mutations is challenging, and further studies are needed to verify how different mutation combinations could lead to distinct clinical and phenotypic profile.

The achievement of CR after induction therapy has been accepted as a precondition for long-term survival. If no CR is achieved after induction therapy, the possible mortality of AML can reach 75% within one year [32]. The CR rate varies across different molecular alteration groups. In our study, *CEBPA* mutations were found to be favorable factors for achieving CR, whereas *ASXL1*, *DNMT3A* and *FLT3-ITD* mutations were unfavorable factors, data consistent with previous reports [19, 33, 34]. Combining all mutation events, Loghavi's group [7, 30, 35] confirmed that *NPM1^mut^*/*FLT3^mut^*/*DNMT3A^mut^* patients seem to have worse clinical outcomes than those with *FLT3^mut^*/*DNMT3A^mut^* and those with *NPM1*^mut^/*DNMT3A*^mut^. In this study, four patients with *NPM1^mut^*/*FLT3-ITD^mut^*/*DNMT3A^mut^* were all non-remission after induction chemotherapy and all died within six months.

The prognostic impact of each mutation and/or co-occurring mutations needs individual and independent clarification. Studies indicate that some mutations have definite prognostic significance and *FLT3-ITD*, *NPM1*, *CEBPA* mutations had been incorporated into the ELN stratification system [2, 36–39]. In this study, *FLT3-ITD*, *ASXL1* and *DNMT3A* mutations were identified to be associated with adverse OS or DFS, while *CEBPA* mutations were associated with favorable prognosis. Although we couldn't ensure their treatment uniform owing to the retrospective study, the treatment between each mutation and wild group for survival analysis was relatively balanced. Due to the limited number of our cases, we didn't further group these patients according to whether receiving HSCT, but the proportion of transplant patients in two groups analyzed for survival analysis was relatively equivalent (Supplementary Table S6). Moreover, we conducted Multivariate COX regression analysis to adjust the impact of HSCT, *FLT3-ITD* and *CEBP*A mutations were identified as independent prognostic factors.

As multiple mutations often coexist in a single patient and more novel mutations were discovered, comprehensive analysis of combined multiple gene mutations is required. However, due to our limited number of cases, we could not scientifically propose a new prognostic model by integrating multiple mutations including those novels. According to the prognostic model recently proposed by Patel's group [4], the intermediate-risk population was reduced by about one-third in our study, thereby guiding different clinical treatment choices for future prospective study. Ultimately, AML treatment may be personalized based on risk stratification. Studies indicated that allo-HSCT can lead to better clinical outcomes for patients with unfavorable-risk cytogenetics in the first CR [40–42]. For patients with adverse-risk genotypes (other than *NPM1^mut^*/*FLT3-ITD^neg^* or mutated *CEBPA*), allo-HSCT in the first CR was also suggested [40, 43–45]. Similarly, in this study, patients with unfavorable molecular genotypes seem to have superior OS when they received allo-HSCT compared to those who did not. For those with intermediate-risk cytogenetics, studies showed that auto-HSCT may represent an alternative therapeutic approach, as some had similar clinical outcomes with allo-HSCT. Still, all of these studies lacked further molecular stratification to the patients [45, 46]. In our study, five patients with intermediate molecular genotypes who received auto-HSCT had similar OS but less transplantation-related morbidity and mortality than those who received allo-HSCT. Prospective studies with more patients are required to verify such observations.

In conclusion, simultaneous screening of multiple genes with high sensitivity and specificity make target sequencing a valuable next-generation sequencer for massive mutation screening of AML samples. Comprehensive genomic analysis based on NGS technology has identified additional novel mutations and several cooperative and exclusive mutation patterns. Elucidation of underlying important relationships among mutated genes and novel pathways provides a theoretical basis for comprehensive understanding of AML biology and molecular pathogenesis. Finally, the relationship between mutations and clinical features such as leukocyte count at diagnosis, immunophenotype and effect assessments can be explored. Integrating data from these multiple sources can increase predictive efficacy for outcomes with traditional treatment to avoid over- or under- treatment. Most importantly, integrated mutational profiling will improve clinical AML management by providing a framework for IR-AML risk stratification and insight into potential therapeutic targets. A proportion of IR-AML defined by cytogenetics alone can be reclassified into favorable or unfavorable risk group according to molecular genotype. More detailed genetic information and large-scale prospective studies are required in the future to evaluate prognosis, predict treatment responses and supervise clinical decisions in AML.

## MATERIALS AND METHODS

### Patients and methods

From September 2008 and March 2014, 95 newly diagnosed *de novo* non-M3 AML patients with IR cytogenetics and healthy adults were recruited for mutational analysis. Patients with antecedent hematological diseases or therapy-related AML were excluded. The diagnosis and classification of AML were based on the FAB Cooperative Group Criteria [47]. Then, 32 peripheral blood samples from the healthy adults were collected as target background control templates. Another 26 bone marrow and matched control saliva samples from newly diagnosed AML were collected to test the reliability of our method to identify somatic mutations. Genomic DNA was extracted from patient bone marrow samples using the standard methods [48]. Metaphase chromosomes were banded by trypsin-Giemsa technique and karyotyped according to the International System for Human Cytogenetic Nomenclature. Flow cytometric analysis was performed on a BD Calibur Flow Cytometer or FC500 Flow Cytometer, and data were analyzed by using Cell Quest or CXP software.

### Target sequencing and variant analysis

All 410 known or putative genes for leukemia were processed for mutations in all IR-AML, 26 newly diagnosed AML and 32 healthy samples by target sequencing. NimbelGen SeqCap EZ Choice was used according to the manufacturer's protocol with modification. Multiplexed libraries were sequenced using 100-bp paired-end runs on an Illumina HiSeq 2500.

Reads were aligned, using Burrows-Wheeler alignment (BWA) tool, to human genomic reference sequences (HG19, NCBI built 37) [49]. To identify of SNPs and INDELs, GATK with recommended parameters was performed [50], Pindel (0.2.4) was performed to identify the *FLT3* internal tandem duplications (*ITD*) [51]. All mutations were annotated by ANNOVAR software using some resources (details were in Supplementary Method) [52]. For 26 newly diagnosed AML samples, two methods were used to identify somatic mutations. The method described was applied in the absence of matched saliva samples, and VarScan with the default parameters was used in the present of matched saliva samples. Somatic mutations identified from the two methods were compared. A subset of somatic mutations detected by target sequencing was independently confirmed by Sanger sequencing.

### Cell line dilution

To determine the effect of varying tumor content on the ability of target-Seq to detect SNP, cells from an AML1-ETO fusion-positive Kasumi-1 cell line was diluted with cells from an BCR-ABL fusion-positive K562 cell line to decrease Kasumi-1 cell content as follows 100, 50, 20, 10, 5, 3, 1, 0.5, 0.3 and 0% (Supplementary Table S7). Cells were resuspended in aliquots of 1×10^6^/ml. A total of 1μg DNA from each of these dilutions was used for library construction.

### Statistical analysis

A Mann-Whitney *U*-test was used to compare the differences in continuous variables. Analysis of frequencies was performed using Fisher's exact test or Pearson's χ^2^ test for larger tables. The logistic regression model was used to identify risk factors for achieving CR. Survival analysis was performed by the Kaplan-Meier method and a Cox proportional hazards model was used to assess the prognostic significance of the clinical variables. OS was defined as the time from diagnosis to death from any cause or last follow-up. DFS was defined as the time from the day achieving CR to the date of relapse, death, or last follow-up. Patients undergoing HSCT were not censored at the time of transplantation. A two-sided *p*-value< 0.05 was considered statistically significant. All statistical analyses were performed with the SPSS 19.0.

## SUPPLEMENTARY METHODS, FIGURES AND TABLES










