# Supplementary material for: Mutational spectrum and risk stratification of intermediate-risk acute myeloid leukemia patients based on next-generation sequencing
Source: Oncotarget. 2016 Jan 27;7(22):32065–78. doi: 10.18632/oncotarget.7028 (PMC5077997; doi:10.18632/oncotarget.7028)
Supplement: Supplementary file 1 [file oncotarget-07-32065-s001.pdf]

Mutational spectrum and risk stratification of intermediate-risk acute myeloid leukemia patients based on next-generation sequencing

SUPPLEMENTARY METHODS

Illumina library construction and sequencing

Indexed Illumina NGS libraries were prepared from 95 IR-AML bone marrow samples, 26 newly diagnosed AML with matched control samples, and 32 peripheral blood leukocyte genomic DNA samples. For these cases, 1ug DNA was sheared before library construction with a Bioruptor UCD-600 (NGS) (Diagenod) instrument using the recommended settings for 200-bp fragments. NGS libraries were constructed following the Illumina standard DNA sample preparation protocol using KAPA HiFi DNA Polymerase (a DNA polymerase possessing strong 3′→5′ exonuclease activity and displaying the lowest published error rate). Library purity and concentration was assessed with a Qubit2.0 Fluorometer (Invitrogen). Fragment length was measured on a 2100 Bioanalyzer using the DNA 1000 Kit (Agilent).

NimbleGen SeqCap EZ Choice was used according to the manufacturer's protocol with modifications. Between 10-14 indexed Illumina libraries were included in one capture hybridization. After hybrid selection, captured DNA fragments were amplified with 13 cycles of PCR using 1× KAPA HiFi Hot Start Ready Mix and 0.4 μM Illumina backbone oligonucleotides in 50-μL reactions. The reactions were then pooled and quantified with QPCR. Multiplexed libraries were sequenced using 100-bp paired-end runs on an Illumina HiSeq 2500.

Mutation detection pipeline

For 95 IR-AML, the SCARF file was converted to FASTQ format by Casava software version 1.8 (Illumina). Raw sequence reads were filtered with an indigenous program. Reads with more than 5% N bases, or of which at least 50% bases had Q ≤5, were eliminated. The remaining reads were aligned, using a Burrows-Wheeler alignment (BWA-0.7.5a) tool, to human genomic reference sequences (HG19, NCBI built 37) with certain parameters (aln -o 1 -e 63 -i 15 -L -l 31 -k 1 -t 6, sample -a 200) [1]. To decrease PCR duplication bias, the resulting Bam files were processed with Samtools. Only unique reads were delivered for analyses. For identification of SNP and indel, GATK with recommended parameters was performed [2], Pindel (0.2.4) was performed to identify the FLT3 internal tandem duplications (ITD) [3]. All mutations were annotated by ANNOVAR software [4] using the following resources:

1. All annotated transcripts in RefSeq Gene;
2. Known constitutional polymorphisms as reported in known human variation databases, such as 1000 genomes release XX, Exome Aggregation Consortium (ExAC) release XX and dbSNP XX [5];
3. Known somatic variation in myeloid and other malignancies as reported in COSMIC v70 [6];

To identify high-confidence somatic variants in all IR-AML samples in the absence of matched control samples, the following criteria were used:

1. Removal of all variants within intronic, UTR and intergenic regions, and retention of only nonsynonymous, frameshift and stopgain mutations in exonic regions;
2. Removal of known polymorphisms present in either 1000 genome or ExAC databases at a population frequency > 0.005;
3. Removal of all variants represent in at least 1 of 32 healthy individuals or saliva samples in 26 newly diagnosed AML patients;
4. Removal of all variants with one of the following features in GATK results: read depth less than 30, Phred-scaled *p*-value using Fisher's exact test to detect strand bias more than 60, variant confidence/quality by depth below 2, mapping quality lower than 40, Alt/Ref read mapping qualities not more than -12.5 and Alt/Ref read position bias not over -8;
5. Removal of all variants with Allele Frequency >0.42 and <0.58 if the variant was not annotated as somatic in COSMIC database. For variants marked MOSMIC whit Frequency > 0.47 and <0.53 were Removed.

Remove harmless mutations

Because we lacked matched normal samples, somatic mutations could not be selected by comparing tumor and matched normal sample. Thus, a series of steps were used to remove germline mutations and harmless mutations. Mutations were removed unless they satisfied all of the following conditions:

1. The mutation depth was larger than 30;
2. The mutation occurred in an exonic region;
3. The mutation function was not "synonymous SNV";
4. The annotation from ClinVar was not "benign" or the mutation did not appear in a dbSNP135 or 1000 Genomes Project (2012 Feb) database.

Evaluation of sensitivity and specificity of cell line dilution data

To evaluate the GATK pipeline in mixed sample, cell dilution data were used. Assuming that 0 and 100% solutions of Kasumi-1 cells were accurate, we hypothesized that at each dilution of these cells we could calculate true SNPs and false-positive SNPs. To test sensitivity limits, we evaluated another method using VarScan. The reads number that support the mutation was considered to be the only requirement for mutation. We set a cutoff at six reads. Supplementary Figure S4 shows that at 3% 81% homozygous and 52% heterozygous mutations were correctly detected in 800x coverage data.

REFERENCES

1. Li H, Durbin R. Fast and accurate short read alignment with Burrows–Wheeler transform. Bioinformatics, 2009, 25:1754–1760.
2. McKenna A, Hanna M, Banks E, et al. The Genome Analysis Toolkit: a MapReduce framework for analyzing next-generation DNA sequencing data. Genome research, 2010, 20:1297–1303.
3. Ye K, Schulz M H, Long Q, et al. Pindel: a pattern growth approach to detect break points of large deletions and medium sized insertions from paired-end short reads. Bioinformatics, 2009, 25:2865–2871.
4. Wang K, Li M, Hakonarson H. ANNOVAR: functional annotation of genetic variants from high-throughput sequencing data. Nucleic acids research, 2010, 38:e164–e164.
5. Joober R. The 1000 Genomes Project: deep genomic sequencing waiting for deep psychiatric phenotyping. J Psychiatry Neurosci. 2011; 36:147–149.
6. Forbes SA, Bindal N, Bamford S, et al. COSMIC: mining complete cancer genomes in the Catalogue of Somatic Mutations in Cancer. Nucleic Acids Res. 2011; 39:945–950.

SUPPLEMENTARY FIGURES AND TABLES

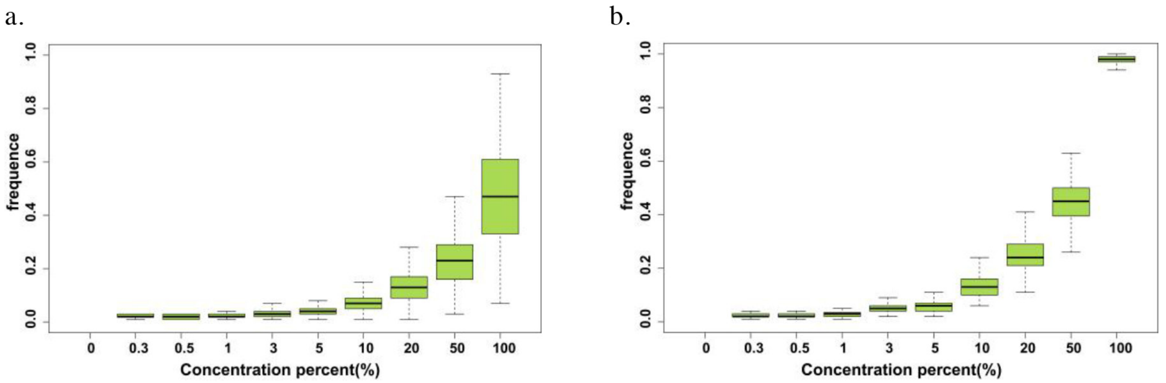

Supplementary Figure S1: Frequency of unique SNP in Kasumi-1 at each level. The distribution of variant allele frequencies of unique SNPs in Kasumi-1 cells at each level. **a.** Heterozygous mutations were extracted to evaluate the relation between variant allele frequency and concentration percent. **b.** Homozygous mutations are shown. The allele frequency of unique SNPs in Kasumi-1 cells reflects Kasumi-1 cell concentration.

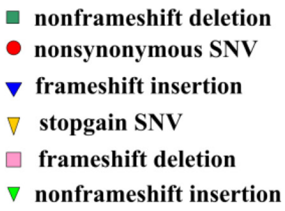

FLT3

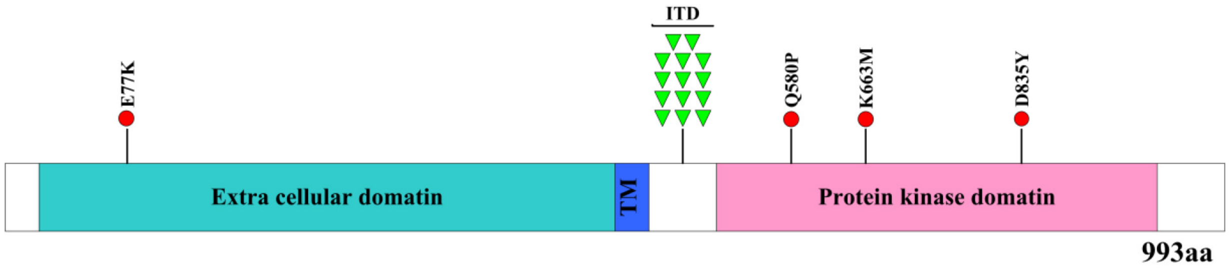

KIT

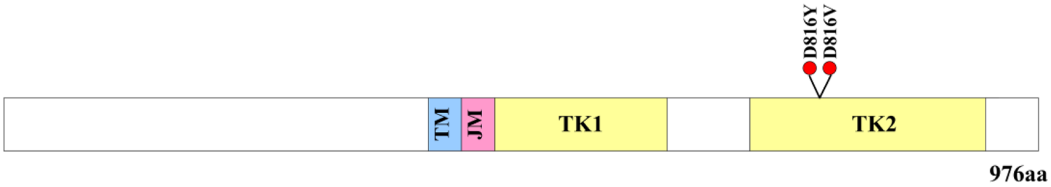

NRAS

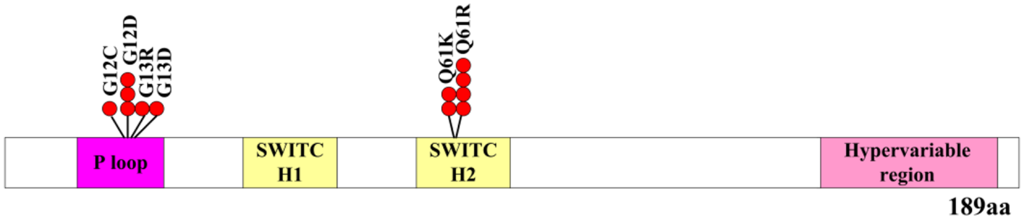

KRAS

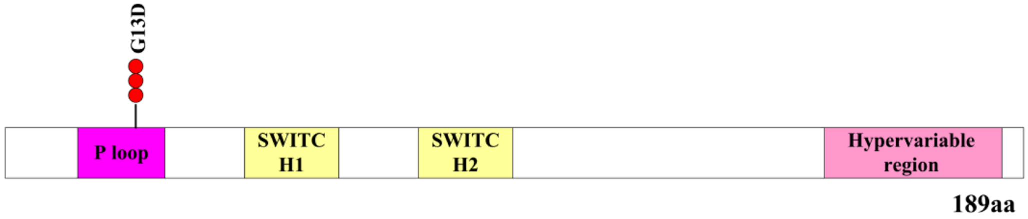

PTPN11

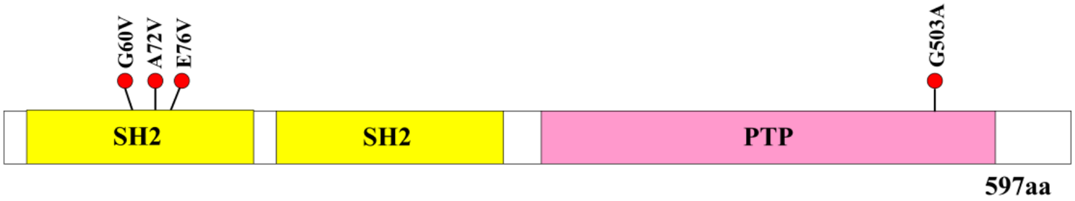

RUNX1

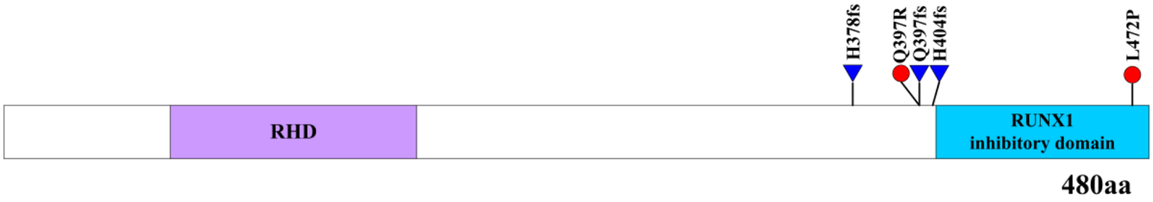

IDH1

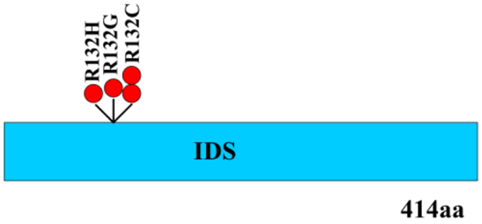

IDH2

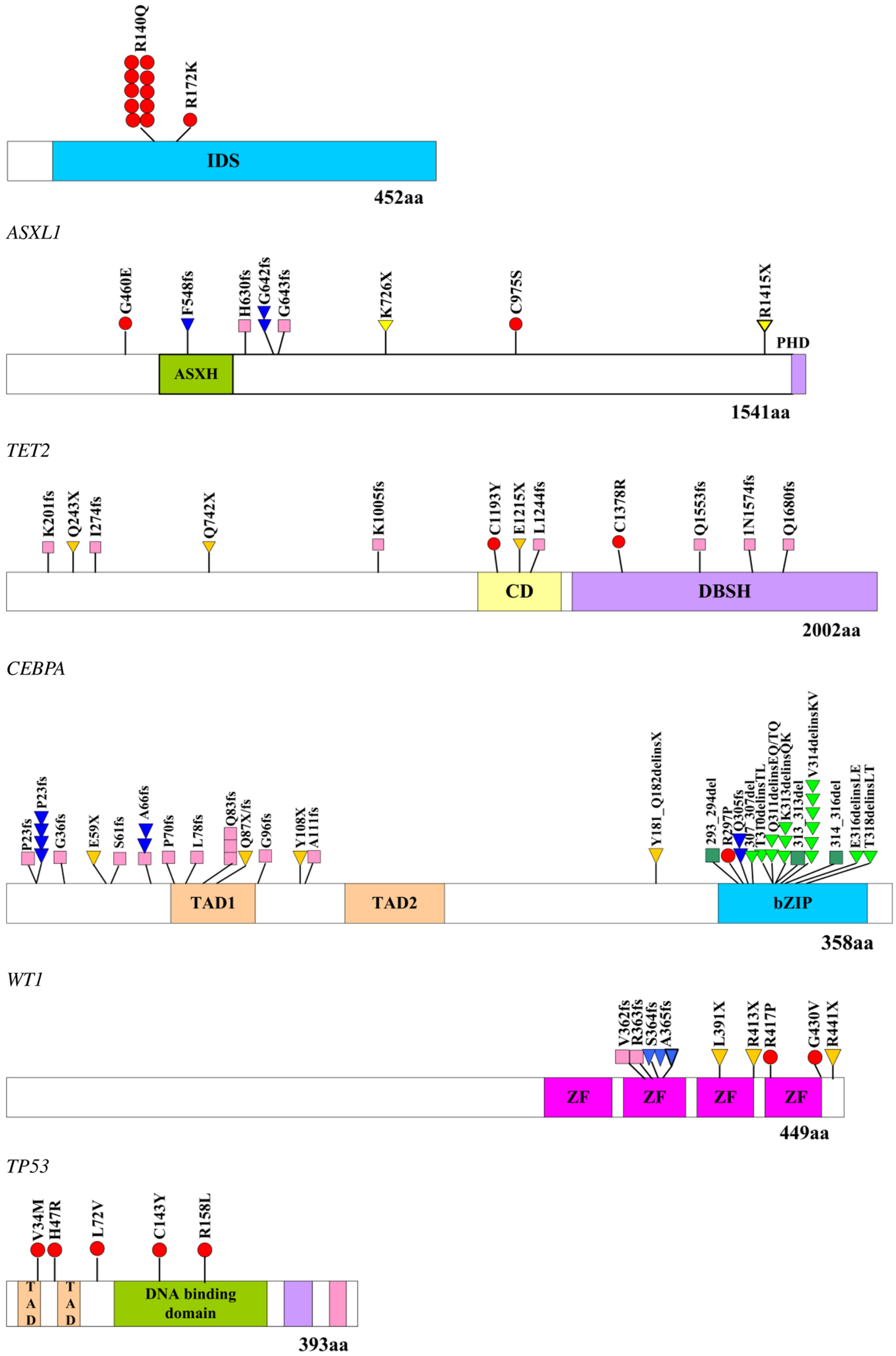
R140Q
R172K
IDS
452aa
ASXL1
G460E
F548fs
H630fs
G642fs
G643fs
K726X
C975S
R1415X
ASXH
PHD
1541aa
TET2
K201fs
Q243X
I274fs
Q742X
K1005fs
C1193Y
E1215X
L1244fs
C1378R
Q1553fs
1N1574fs
Q1680fs
CD
DBSH
2002aa
CEBPA
P23fs
P23fs
G36fs
E59X
S61fs
A66fs
P70fs
L78fs
Q83fs
Q87X/fs
G96fs
Y108X
A111fs
Y181_Q182delinsX
293_294del
R297P
Q305fs
307_307del
T310delinsTL
Q311delinsEQ/TQ
K313delinsQK
313_313del
V314delinsKV
314_316del
E316delinsLE
T318delinsLT
TAD1
TAD2
bZIP
358aa
WT1
V362fs
R363fs
S364fs
A365fs
L391X
R413X
R417P
G430V
R441X
ZF
ZF
ZF
ZF
449aa
TP53
V34M
H47R
L72V
C143Y
R158L
TAD
TAD
DNA binding domain
393aa

DNMT3A

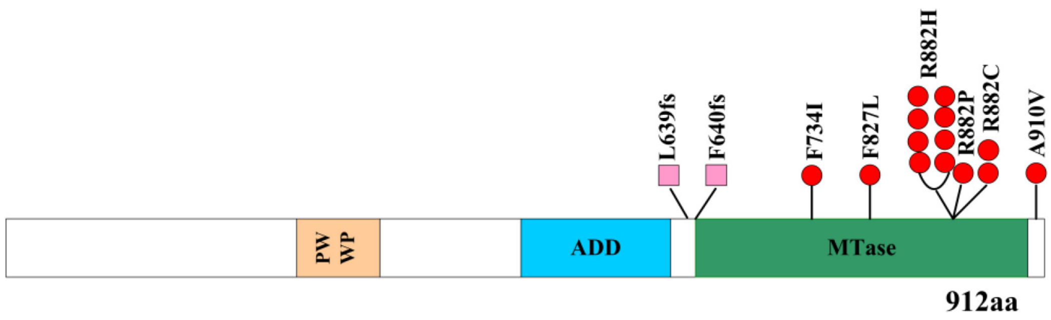

Supplementary Figure S2: Position and type of mutations identified in this study.

Significantly overlapped mutations

	NPM1	
DNMT3A	wt	mut
wt	69	10
mut	6	10
p-value	p=0.000	
	NPM1	
FLT3-ITD	wt	mut
wt	69	12
mut	6	8
p-value	p=0.000	
	FLT3-ITD	
DNMT3A	wt	mut
wt	71	8
mut	10	6
p-value	p=0.005	
	WT1	
NRAS	wt	mut
wt	77	6
mut	8	4
p-value	p=0.024	

Mutually exclusive mutations

	IDH1/2	
TET2	wt	mut
wt	71	15
mut	9	0
p-value	p=0.000	
	WT1	
TET2	wt	mut
wt	76	10
mut	9	0
p-value	p=0.000	
	CEBPA	
NPM1	wt	mut
wt	45	27
mut	20	0
p-value	p=0.000	
	STAG2	
SMC3	wt	mut
wt	88	5
mut	2	0
p-value	p=0.000	

Supplementary Figure S3: Cooperative and exclusive patterns of overlapping mutations. Significantly overlapped mutations were observed among *FLT3-ITD* mutations and *NPM1, DNMT3A* mutations; *WT1* and *NRAS* mutations. In contrast, mutually exclusive mutations were observed between *IDH1/2 and TET2* mutations*; WT1 and TET2* mutations*; CEBPA and NPM1* mutations; and among the Cohesion complex mutations.

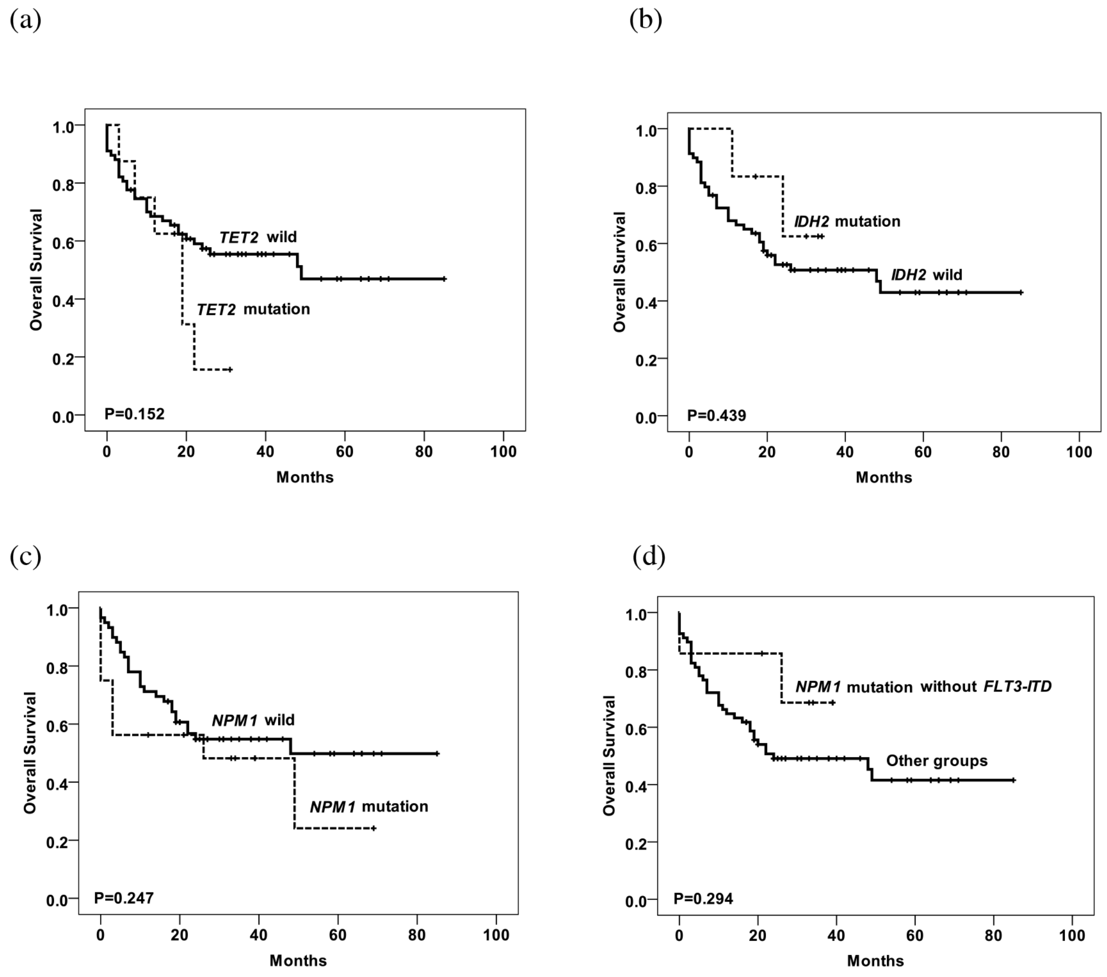

Supplementary Figure S4: Kaplan-Meier curves of overall survival for single-gene mutations. Kaplan-Meier curves according to the mutations are shown. P value was estimated by the log-rank test. The mutated number of *TET2*, *IDH2* and *NPM1* is 9, 11 and 20 respectively. Of which 8, 6 and 16 patients were used for survival analysis, respectively. **a.** Kaplan-Meier curves of overall survival for TET2 mutations. **b.** Kaplan-Meier curves of overall survival for IDH2 mutations. **c.** Kaplan-Meier curves of overall survival for NPM1 mutations. d. Kaplan-Meier curves of overall survival for NPM1 mutations without FLT3-ITD.

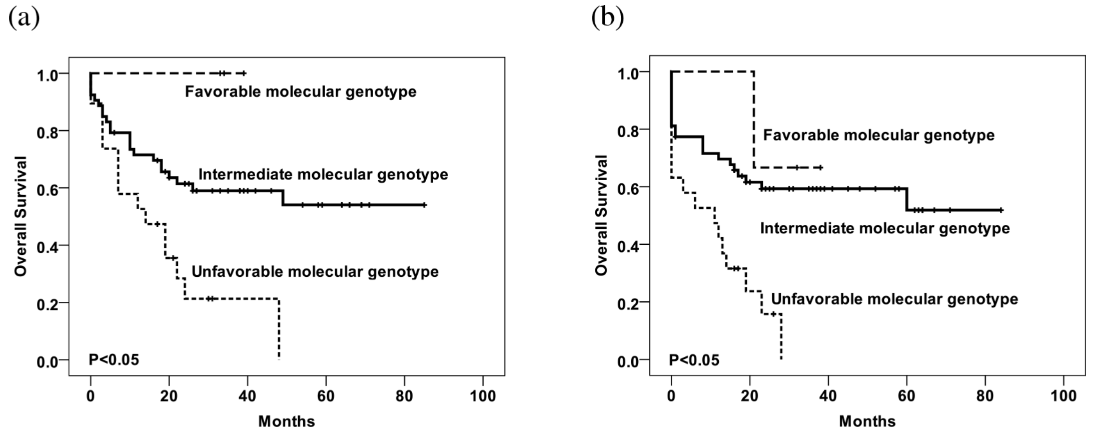

Supplementary Figure S5: Kaplan-Meier curves of OS and DFS for comprehensive mutational analysis according to the model proposed by Patel and colleagues. When the patients were stratified into the risk groups proposed by Patel et al., three distinct prognostic subgroups can be separated, that is, intermediate cytogenetics and favorable genotype, intermediate cytogenetics and genotype, intermediate cytogenetics and unfavorable genotype which included 5, 67 and 23 patients, respectively. Of which 3, 53 and 19 patients were used for survival analysis, respectively. **a.** Kaplan-Meier curves of OS. **b.** Kaplan-Meier curves of DFS.

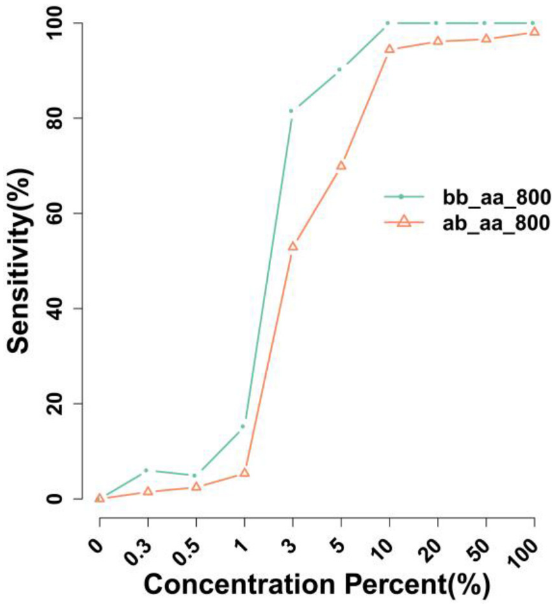

Supplementary Figure S6: Another method to detect SNPs in diluted cells. To measure mutations in cell line dilutions, 800x depth data was used to evaluate performance. At 3%, 81% homozygous mutations and 52% heterozygous mutations can be detected correctly in 800x coverage data.

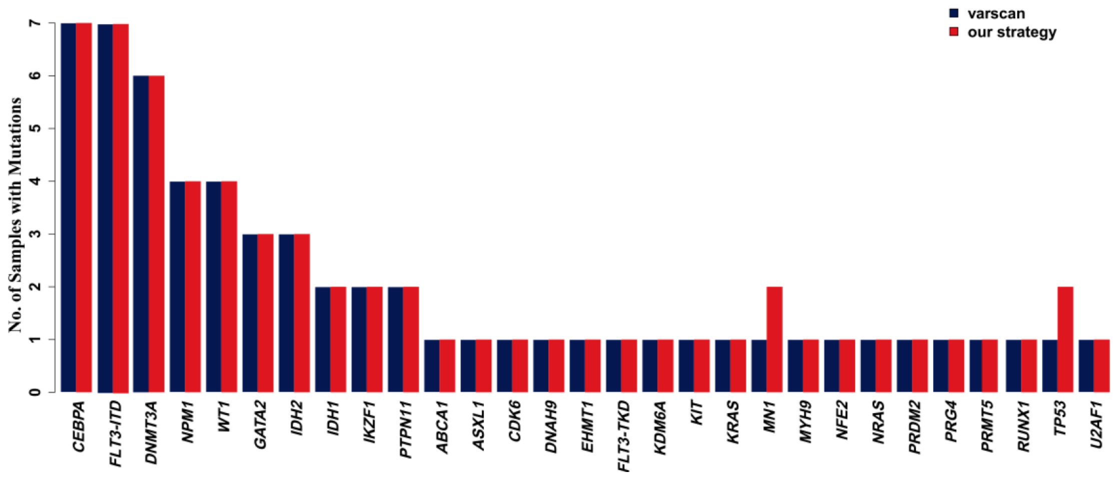

Supplementary Figure S7: Comparison of somatic mutations identified by our strategy and Varscan in 26 newly diagnosed AML patients.

Supplementary Table S1: 410 gene list in this panel.

See Supplementary File 1

Supplementary Table S2: 101 genes identified in this study.

See Supplementary File 2

Supplementary Table S3: Validation of gene mutations by sanger sequencing.

See Supplementary File 3

Supplementary Table S4: The clinical features of single mutational analysis.

See Supplementary File 4

Supplementary Table S5: Clinical features of comprehensive analysis of multiple mutations

Variable	WBC count at diagnosis	*P* value
NMP1^{mut}/*FLT3-ITD*^{mut}/*DNMT3A*^{mut}	132.39(70-332.77)	0.047
NMP1^{mut}/*FLT3-ITD*^{neg}/*DNMT3A*^{mut}	18.01(11.57-135.16)	
**Variable**	**WBC count at diagnosis**	***P* value**
NMP1^{mut}/*FLT3-ITD*^{mut}/*DNMT3A*^{mut}	132.39(70-332.77)	0.047
NPM1^{neg}/*FLT3-ITD*^{neg}/*DNMT3A*^{mut}	21.33(1.78-168.7)	

NMP1^{mut}/*FLT3-ITD*^{mut}/*DNMT3A*^{mut} AML had greater leukocyte counts than *NPM1*^{wt}/*FLT3-ITD*^{neg}/*DNMT3A*^{mut} or *NMP1*^{mt}/*FLT3-ITD*^{neg}/*DNMT3A*^{mut} group. Differences were using a Mann-Whitney *U*-test.

Supplementary Table S6: The proportion of transplant patients in two groups analyzed for survival analysis

Gene	Numbers and proportion of transplant patients in mutation group	Numbers and proportion of transplant patients in non-mutation group
ASXL1	2/6 (33.3%)	36/ 69 (52.1%)
DNMT3A	4/12 (33.3%)	34/63 (53.9%)
FLT3-ITD	7/14 (50.0%)	31/61 (50.8%)
CEBPA	13/22 (59.0%)	25/53 (47.2%)
NPM1	8/16 (50.0%)	30/59 (50.8%)

Supplementary Table S7: Cell line dilution data

ID	Component	
	Kasumi-1 (AML1-ETO)	K562 (BCR-ABL)
HC14AN00001	0%	100%
HC14AN00002	3%	99.70%
HC14AN00003	5%	99.50%
HC14AN00004	1%	99%
HC14AN00005	3%	97%
HC14AN00006	5%	95%
HC14AN00007	10%	90%
HC14AN00008	20%	80%
HC14AN00009	50%	50%
HC14AN00010	100%	0%

To evaluate the performance of a mutation detection method, Kasumi-1 and K56 cells were at different ration.
