# Supplementary material for: Mutational spectrum and risk stratification of intermediate-risk acute myeloid leukemia patients based on next-generation sequencing
Source: Oncotarget. 2016 Jan 27;7(22):32065–78. doi: 10.18632/oncotarget.7028 (PMC5077997; doi:10.18632/oncotarget.7028)
Supplement: Supplementary file 2 [file oncotarget-07-32065-s002.docx]

**Table S1: 410 gene list in this panel**

| **DNA Gene List: Entire Coding Sequence** | | | | | | | |
| --- | --- | --- | --- | --- | --- | --- | --- |
| *ABCA1* | *CAV1* | *ELN* | *HOXC11* | *KMT1D* | *MPL* | *PIM1* | *TAF15* |
| *ABCA12* | *CBFA2T1* | *EP300* | *HOXC13* | *SETDB1* | *MPV17* | *PKD1L2* | *TAL1* |
| *ABCA2* | *CBFA2T3* | *EPS15* | *HOXD11* | *KMT1F* | *MSF* | *PML* | *TAL2* |
| *ABCA4* | *CBFB* | *ERG* | *HOXD13* | *MLL1* | *MSH4* | *PMS1* | *TBET* |
| *ABCA7* | *CBL* | *ESR1* | *ICAM1* | *MLL2* | *MSI2* | *PNUTL1* | *TCF3* |
| *ABCB4* | *CBLB* | *ETS1* | *ICSBP* | *MLL3* | *MSN* | *POU2AF1* | *TCL1A* |
| *ABCB7* | *CBLC* | *ETV6* | *ID2* | *MLL4* | *MTCP1* | *PRAME* | *TCL1B* |
| *ABCC2* | *CREBBP* | *EVI1* | *ID3* | *MLL5* | *MUC1* | *PRDM16* | *NR4A2* |
| *ABCC6* | *CCDC6* | *EVI2A* | *ID4* | *SET1A* | *MYC* | *PRDM3* | *TCL6* |
| *ABCC8* | *CCDC88C* | *EVI2B* | *IDH1* | *SET1B* | *MYD88* | *PRF1* | *TET1* |
| *ABCC9* | *CCND1* | *EWSR1* | *IDH2* | *ASH1* | *MYH11* | *PRMT1* | *TET2* |
| *ABCD1* | *CCND2* | *EZH1* | *IFNG* | *SET* | *MYH9* | *PRMT4* | *TFG* |
| *ABCD3* | *CD28* | *EZH2* | *IKAROS* | *NSD1* | *MYO18A* | *PRMT5* | *TFPT* |
| *ABCG5* | *CD44* | *FACL6* | *IKZF1* | *SMYD2* | *MYST4* | *PRMT7* | *THAP1* |
| *ABCG8* | *CD48* | *FAM5C* | *IL-1* | *DOT1L* | *NCOA2* | *PRTN3* | *THRA* |
| *ABL1* | *CD58* | *FANCA* | *IL-2* | *KMT5A* | *NDE1* | *PTCH1* | *TIF1* |
| *ABL2* | *CD80* | *FBXW7* | *IL-3* | *KMT5B* | *NF1* | *PTEN* | *TLR1* |
| *MLLT10* | *CD86* | *FCGR2B* | *IL-4* | *KMT5C* | *NF-E2* | *PTPN11* | *TLR2* |
| *AF15Q14* | *CDC23* | *FGFR1* | *IL-6* | *KMT7* | *NFKB2* | *PTPRD* | *TLR3* |
| *AF1Q* | *CDK5RAP2* | *FGFR1OP* | *IL-10* | *KMT8* | *NIN* | *PTPs* | *TLR4* |
| *AF3p21* | *CDK6* | *FGFR1OP2* | *IL-15* | *KRAS* | *NOTCH1* | *RABEP1* | *TLR9* |
| *AF5q31* | *CDX2* | *FGFR3* | *IL-18* | *KRAS2* | *NOTCH3* | *RAD21* | *TLX1* |
| *ALK* | *CEBPA* | *FIP1L1* | *IL-21* | *LAF4* | *NPM1* | *RAD50* | *TLX3* |
| *RNF213* | *CHK2* | *FLT3* | *IL-27* | *LASP1* | *NR4A3* | *RAP1GDS1* | *TNF* |
| *ANKRD24* | *CISH* | *FNBP1* | *IRF1* | *LCK* | *NRAS* | *RARA* | *TNFAIP3* |
| *ARHGEF* | *CLC* | *FOXO3A* | *IRF8* | *LCP1* | *NRG3* | *RB1* | *TNFRSF17* |
| *ARNT* | *CLTC* | *FOXP1* | *IRTA1* | *LCX* | *NSD1* | *RECQL4* | *TOP1* |
| *ASXL1* | *CLTCL1* | *FSTL3* | *ITGA2* | *LDB1* | *NSD2* | *RGS2* | *TP53* |
| *ATIC* | *C-MAF* | *FUS* | *ITGB1* | *LMO1* | *NSD3* | *RNU6-19* | *TP53BP1* |
| *ATM* | *CNTRL* | *FVT1* | *ITGB2* | *LMO2* | *NTRK1* | *RPN1* | *TPM3* |
| *AXL* | *COL1A1* | *GAS7* | *ITK* | *LOC* | *NUP214* | *RUNX1* | *TPM4* |
| *BAALC* | *CPSF6* | *GATA1* | *JAK1* | *LPP* | *NUP98* | *RUNX1T1* | *TRIP11* |
| *BAX* | *CRBN* | *GATA2* | *JAK2* | *LYL1* | *Nut* | *RUNXBP2* | *TTL* |
| *BCL10* | *CREBBP* | *GATA3* | *JAK3* | *MAFB* | *OMD* | *RYR1* | *U2AF1* |
| *BCL11A* | *CRLF2* | *GIT2* | *JMJD6* | *MAGEA1* | *PADI4* | *SELL* | *U2AF2* |
| *BCL11B* | *CSF3R* | *GMPS* | *KDM1* | *MALT1* | *PAFAH1B2* | *SEPT6* | *UTX* |
| *BCL2* | *CXCR4* | *GOLGA4* | *KDM2A* | *MDS1* | *PAX7* | *SET* | *VHL* |
| *BCL3* | *D10S170* | *GPHN* | *KDM2B* | *MECOM* | *PBX1* | *SETBP1* | *WDR48* |
| *BCL6* | *DDX10* | *GRAF* | *KDM3A* | *MKL1* | *PBX2* | *SF3B1* | *WHSC1L1* |
| *BCL7A* | *DDX4* | *HEAB* | *KDM3B* | *MLF1* | *PBX3* | *SH3GL1* | *WT1* |
| *BCL9* | *DDX6* | *HIP1* | *KDM4A* | *MLL* | *PCM1* | *SIL* | *ZBTB16* |
| *BCR* | *DEK* | *HLA-E* | *KDM4B* | *MLL3* | *PCSK7* | *SMC1A* | *ZMYM2* |
| *BIN2* | *DIS3* | *HLA-G* | *KDM5A* | *MLLT1* | *PDCD1* | *SMC3* | *ZNF145* |
| *BIRC3* | *DLEU1* | *HLF* | *KDM5B* | *MLLT10* | *PDCD10* | *SPECC1* | *ZNF384* |
| *BIRC5* | *DNAH9* | *HLXB9* | *KDM5C* | *MLLT2* | *PDGFRA* | *SPHK1* | *ZNF521* |
| *BRAF* | *DNMT1* | *HNRNPK* | *KDM5D* | *MLLT3* | *PDGFRB* | *SPRY3* | *ZNFN1A1* |
| *BRCA1* | *DNMT3A* | *HOX11* | *KDM6A* | *MLLT4* | *PER1* | *SPRY4* |  |
| *BTG1* | *DNMT3B* | *HOX11L2* | *KDM6B* | *MLLT6* | *PHF5* | *SRSF2* |  |
| *CACNA1E* | *E2F4* | *HOXA10* | *KIT* | *MLLT7* | *PHF6* | *SSH3BP1* |  |
| *CAPRIN1* | *EGR1* | *HOXA11* | *KMT1A* | *MN1* | *PICALM* | *STAG2* |  |
| *CARD11* | *ELF4* | *HOXA13* | *KMT1B* | *MORF* | *PICH* | *STIL* |  |
| *CARS* | *ELL* | *HOXA9* | *KMT1C* | *MOZ* | *PIK3CA* | *STRN* |  |
