# Supplementary material for: Mutational spectrum and risk stratification of intermediate-risk acute myeloid leukemia patients based on next-generation sequencing
Source: Oncotarget. 2016 Jan 27;7(22):32065–78. doi: 10.18632/oncotarget.7028 (PMC5077997; doi:10.18632/oncotarget.7028)
Supplement: Supplementary file 3 [file oncotarget-07-32065-s003.docx]

**Table S2: 101 genes identified in this study**

| **Sample ID** | **Gene** | **Chrom** | **Nucleotide**  **change** | **Amino acid**  **change** | **Refseq** |
| --- | --- | --- | --- | --- | --- |
| D-2860 | *ABCA2* | chr9 | c.275C>G | p.T92R | NM_001606 |
| D-2978 | *ABCC9* | chr12 | c.2530A>G | p.I844V | NM_005691 |
| D-2964 | *ABCC9* | chr12 | NA | NA | NM_005691 |
| D-2997 | *ABCG8* | chr2 | c.211T>A | p.F71I | NM_022437 |
| D-3007 | *ASCL1* | chr12 | c.149_150ins | p.A50delins | NM_004316 |
| D-2821 | *ASCL1* | chr12 | c.150_164del | p.50_55del | NM_004316 |
| D-2077 | *ASXL1* | chr20 | c.1379G>A | p.G460E | NM_015338 |
| D-2084 | *ASXL1* | chr20 | c.1927delG | p.G643fs | NM_015338 |
| D-2861 | *ASXL1* | chr20 | c. 1927dupG | p.G642fs | NM_015338 |
| D-2957 | *ASXL1* | chr20 | c. 1927dupG | p.G642fs | NM_015338 |
| D-2961 | *ASXL1* | chr20 | c.1888_1910del | p.H630fs | NM_015338 |
| D-2963 | *ASXL1* | chr20 | c.1643_1644ins | p.F548fs | NM_015338 |
| D-2841 | *ASXL1* | chr20 | c.2176A>T | p.K726X | NM_015338 |
| D-2078 | *ASXL1* | chr20 | c.2924G>C | p.C975S | NM_015338 |
| D-2966 | *ASXL1* | chr20 | c.4243C>T | p.R1415X | NM_015338 |
| D-2838 | *ATM* | chr11 | c.274A>G | p.K92E | NM_000051 |
| D-2850 | *ATM* | chr11 | c.146C>G | p.S49C | NM_000051 |
| D-2999 | *ATM* | chr11 | c.283C>A | p.Q95K | NM_000051 |
| D-2966 | *ATM* | chr11 | c.2537T>C | p.L846P | NM_000051 |
| D-2826 | *ATM* | chr11 | c.2540T>C | p.M847T | NM_000051 |
| D-2976 | *BAX* | chr19 | c.118C>T | p.R40X | NM_138763 |
| D-2843 | *BIRC5* | chr17 | c.383G>A | p.S128N | NM_001012270 |
| D-2862 | *BRINP3* | chr1 | c.52T>C | p.W18R | NM_199051 |
| D-2974 | *BTG1* | chr12 | c.116C>A | p.T39N | NM_001731 |
| D-2822 | *C19orf10* | chr19 | c.182A>G | p.Y61C | NM_019107 |
| D-2987 | *CACNA1E* | chr1 | c.1114C>T | p.R372C | NM_000721 |
| D-2961 | *CBLB* | chr3 | c.2936G>A | p.R979H | NM_170662 |
| D-2989 | *CBLB* | chr3 | c.2556T>G | p.F852L | NM_170662 |
| D-2853 | *CCDC88C* | chr14 | c.3803T>A | p.L1268Q | NM_001080414 |
| D-2953 | *CCDC88C* | chr14 | c.6026C>T | p.P2009L | NM_001080414 |
| D-2954 | *CCDC88C* | chr14 | c.1878G>C | p.K626N | NM_001080414 |
| D-2977 | *CD28* | chr2 | c.236G>A | p.R79H | NM_001243078 |
| D-2978 | *CD28* | chr2 | c.298C>T | p.R100C | NM_001243078 |
| D-2864 | *CD44* | chr11 | c.968C>G | p.P323R | NM_001001389 |
| D-2083 | *CEBPA* | chr19 | c.951_952insCTG | p.T318delinsLT | NM_004364 |
| D-2851 | *CEBPA* | chr19 | c.941_946del | p.314_316del | NM_004364 |
| D-2979 | *CEBPA* | chr19 | c.945_946insCTG | p.E316delinsLE | NM_004364 |
| D-2983 | *CEBPA* | chr19 | c.937_939del | p.313_313del | NM_004364 |
| D-2085 | *CEBPA* | chr19 | c.939_940insAAG | p.V314delinsKV | NM_004364 |
| D-2832 | *CEBPA* | chr19 | c.939_940insAAG | p.V314delinsKV | NM_004364 |
| D-2858 | *CEBPA* | chr19 | c.939_940insAAG | p.V314delinsKV | NM_004364 |
| D-2970 | *CEBPA* | chr19 | c.939_940insAAG | p.V314delinsKV | NM_004364 |
| D-2975 | *CEBPA* | chr19 | c.939_940insAAG | p.V314delinsKV | NM_004364 |
| D-2986 | *CEBPA* | chr19 | c.939_940insAAG | p.V314delinsKV | NM_004364 |
| D-2073 | *CEBPA* | chr19 | c.936_937insCAG | p.K313delinsQK | NM_004364 |
| D-2990 | *CEBPA* | chr19 | c.936_937insCAG | p.K313delinsQK | NM_004364 |
| D-3006 | *CEBPA* | chr19 | c.936_937insCAG | p.K313delinsQK | NM_004364 |
| D-2955 | *CEBPA* | chr19 | c.917_934del | p.306_312del | NM_004364 |
| D-2830 | *CEBPA* | chr19 | c.930_931insACG | p.Q311delinsTQ | NM_004364 |
| D-2967 | *CEBPA* | chr19 | c.929_930insTCT | p.T310delinsTL | NM_004364 |
| D-3120 | *CEBPA* | chr19 | c.919_921del | p.307_307del | NM_004364 |
| D-2863 | *CEBPA* | chr19 | c.919_921linsAGGC | p.N307dellinsKGH | NM_004364 |
| D-2821 | *CEBPA* | chr19 | c.914_915insAGGA | p.Q305fs | NM_004364 |
| D-2821 | *CEBPA* | chr19 | c.912_913insGC | p.Q305fs | NM_004364 |
| D-2969 | *CEBPA* | chr19 | c.878_880del | p.293_294del | NM_004364 |
| D-2996 | *CEBPA* | chr19 | c.542dupA | p.Y181_Q182delinsX | NM_004364 |
| D-2821 | *CEBPA* | chr19 | c.332_339del | p.A111fs | NM_004364 |
| D-2846 | *CEBPA* | chr19 | c.324 C>A | p.Y108X | NM_004364 |
| D-2981 | *CEBPA* | chr19 | c.286_296del | p.G96fs | NM_004364 |
| D-2085 | *CEBPA* | chr19 | c. 259C>T | p.Q87X | NM_004364 |
| D-2830 | *CEBPA* | chr19 | c.247_256del | p.Q83fs | NM_004364 |
| D-2083 | *CEBPA* | chr19 | c.247delC | p.Q83fs | NM_004364 |
| D-2986 | *CEBPA* | chr19 | c.247delC | p.Q83fs | NM_004364 |
| D-2990 | *CEBPA* | chr19 | c.232delC | p.L78fs | NM_004364 |
| D-2971 | *CEBPA* | chr19 | c.209delC | p.P70fs | NM_004364 |
| D-2838 | *CEBPA* | chr19 | c.196_197insT | p.A66fs | NM_004364 |
| D-2863 | *CEBPA* | chr19 | c.196_197insTAGG | p.A66fs | NM_004364 |
| D-2983 | *CEBPA* | chr19 | c.196delG | p.A66fs | NM_004364 |
| D-3006 | *CEBPA* | chr19 | c.183delC | p.S61fs | NM_004364 |
| D-2073 | *CEBPA* | chr19 | c.175G>T | p.E59X | NM_004364 |
| D-2953 | *CEBPA* | chr19 | c.890G>C | p.R297P | NM_004364 |
| D-2975 | *CEBPA* | chr19 | c.107delG | p.G36fs | NM_004364 |
| D-2076 | *CEBPA* | chr19 | c.68_78del | p.P23fs | NM_004364 |
| D-2851 | *CEBPA* | chr19 | c.68dupC | p.P23fs | NM_004364 |
| D-2955 | *CEBPA* | chr19 | c.68dupC | p.P23fs | NM_004364 |
| D-2970 | *CEBPA* | chr19 | c.68dupC | p.P23fs | NM_004364 |
| D-3120 | *CEBPA* | chr19 | c.68dupC | p.P23fs | NM_004364 |
| D-2988 | *CXCR4* | chr2 | c. 685T>A | p.S229T | NM_003467 |
| D-2962 | *DNMT1* | chr19 | c.879_880ins | p.A294_A295delins | NM_001379 |
| D-2962 | *DNMT1* | chr19 | c.445_446ins | p.P149_E150delins | NM_001379 |
| D-3009 | *DNMT3A* | chr2 | c.2162C>T | A721V | NM_153759 |
| D-2081 | *DNMT3A* | chr2 | c.2645G>A | p.R882H | NM_175629 |
| D-2842 | *DNMT3A* | chr2 | c.2645G>A | p.R882H | NM_175629 |
| D-2856 | *DNMT3A* | chr2 | c.2645G>A | p.R882H | NM_175629 |
| D-2861 | *DNMT3A* | chr2 | c.2645G>A | p.R882H | NM_175629 |
| D-2961 | *DNMT3A* | chr2 | c.2645G>A | p.R882H | NM_175629 |
| D-2968 | *DNMT3A* | chr2 | c.2645G>A | p.R882H | NM_175629 |
| D-2985 | *DNMT3A* | chr2 | c.2645G>A | p.R882H | NM_175629 |
| D-3128 | *DNMT3A* | chr2 | c.2645G>A | p.R882H | NM_175629 |
| D-2978 | *DNMT3A* | chr2 | c.2645G>C | p.R882P | NM_175629 |
| D-2857 | *DNMT3A* | chr2 | c.2644C>T | p.R882C | NM_175629 |
| D-2959 | *DNMT3A* | chr2 | c.2644C>T | p.R882C | NM_175629 |
| D-2987 | *DNMT3A* | chr2 | c.2481C>A | p.F827L | NM_175629 |
| D-2964 | *DNMT3A* | chr2 | c.2200T>A | p.F734I | NM_175629 |
| D-2083 | *DNMT3A* | chr2 | c.1920_1923del | p.F640fs | NM_175629 |
| D-2855 | *DNMT3A* | chr2 | NA | NA | NM_175629 |
| D-2855 | *DNMT3A* | chr2 | c.1916delT | p.L639fs | NM_175629 |
| D-2989 | *DNMT3B* | chr20 | c.1596T>G | p.C532W | NM_006892 |
| D-3118 | *DNMT3B* | chr20 | c.1804G>A | p.V602I | NM_006892 |
| D-2996 | *DOT1L* | chr19 | c.1455G>C | p.Q485H | NM_032482 |
| D-2865 | *DOT1L* | chr19 | c.3023C>T | p.S1008F | NM_032482 |
| D-2832 | *DOT1L* | chr19 | c.3050A>G | p.Q1017R | NM_032482 |
| D-2857 | *DOT1L* | chr19 | c.4229C>G | p.A1410G | NM_032482 |
| D-2970 | *EHMT1* | chr9 | c. 275C>T | p.A92V | NM_024757 |
| D-2854 | *ESR1* | chr6 | c.1026G>A | p.M342I | NM_000125 |
| D-2966 | *ETV6* | chr12 | c.346delC | p.L116fs | NM_001987 |
| D-2966 | *ETV6* | chr12 | c.348_352del | p.L116fs | NM_001987 |
| D-2959 | *EVI2A* | chr17 | c.104T>C | p.L35P | NM_014210 |
| D-2990 | *FANCA* | chr16 | c.3532G>A | p.E1178K | NM_000135 |
| D-2982 | *FANCA* | chr16 | c.2291G>A | p.R764Q | NM_000135 |
| D-2848 | *FANCA* | chr16 | c.356C>G | p.S119C | NM_000135 |
| D-2966 | *FBXW7* | chr4 | c.535C>T | p.R179C | NM_033632 |
| D-2864 | *FGFR3* | chr4 | c.1211A>G | p.K404R | NM_000142 |
| D-2085 | *FIP1L1* | chr4 | c.34G>C | p.E12Q | NM_030917 |
| D-2087 | *FLT3* | chr13 | c.2503G>T | p.D835Y | NM_004119 |
| D-2860 | *FLT3* | chr13 | c.1988A>T | p.K663M | NM_004119 |
| D-3007 | *FLT3* | chr13 | c.1739A>C | p.Q580P | NM_004119 |
| D-2960 | *FLT3* | chr13 | c.229G>A | p.E77K | NM_004119 |
| D-2854 | *FNBP1* | chr9 | c.1406G>A | p.R469Q | NM_015033 |
| D-2862 | *FNBP1* | chr9 | c. 301G>T | p.D101Y | NM_015033 |
| D-3007 | *FOXO4* | chrX | c. 902A>G | p.N301S | NM_005938 |
| D-2828 | *FOXP1* | chr3 | c.159C>A | p.H53Q | NM_032682 |
| D-2846 | *GAS7* | chr17 | c.223G>A | p.G75S | NM_003644 |
| D-2828 | *GOLGA4* | chr3 | c.1046G>A | p.R349H | NM_002078 |
| D-2977 | *GOLGA4* | chr3 | c.2192A>T | p.H731L | NM_002078 |
| D-2844 | *GOLGA4* | chr3 | c.3277G>C | p.E1093Q | NM_002078 |
| D-2071 | *HOXA9* | chr7 | c.377C>G | p.S126C | NM_152739 |
| D-2072 | *HOXD11* | chr2 | c.734G>A | p.G245D | NM_021192 |
| D-2988 | *HOXD11* | chr2 | c.734G>A | p.G245D | NM_021192 |
| D-2953 | *HOXD13* | chr2 | c.168_179del | p.56_60del | NM_000523 |
| D-2969 | *ID4* | chr6 | c.379C>G | p.P127A | NM_001546 |
| D-2857 | *IDH1* | chr2 | c.395G>A | p.R132H | NM_005896 |
| D-2843 | *IDH1* | chr2 | c.394C>G | p.R132G | NM_005896 |
| D-2965 | *IDH1* | chr2 | c.394C>T | p.R132C | NM_005896 |
| D-2987 | *IDH1* | chr2 | c.394C>T | p.R132C | NM_005896 |
| D-2957 | *IDH2* | chr15 | c.515G>A | p.R172K | NM_002168 |
| D-2842 | *IDH2* | chr15 | c.419G>A | p.R140Q | NM_002168 |
| D-2848 | *IDH2* | chr15 | c.419G>A | p.R140Q | NM_002168 |
| D-2861 | *IDH2* | chr15 | c.419G>A | p.R140Q | NM_002168 |
| D-2862 | *IDH2* | chr15 | c.419G>A | p.R140Q | NM_002168 |
| D-2954 | *IDH2* | chr15 | c.419G>A | p.R140Q | NM_002168 |
| D-2961 | *IDH2* | chr15 | c.419G>A | p.R140Q | NM_002168 |
| D-2968 | *IDH2* | chr15 | c.419G>A | p.R140Q | NM_002168 |
| D-2981 | *IDH2* | chr15 | c.419G>A | p.R140Q | NM_002168 |
| D-2989 | *IDH2* | chr15 | c.419G>A | p.R140Q | NM_002168 |
| D-2996 | *IDH2* | chr15 | c.419G>A | p.R140Q | NM_002168 |
| D-2863 | *IKZF1* | chr7 | c.472G>A | p.G158S | NM_006060 |
| D-2843 | *ITGA2* | chr5 | c.89A>C | p.Y30S | NM_002203 |
| D-2976 | *ITGA2* | chr5 | c.2269C>T | p.R757C | NM_002203 |
| D-2969 | *JAK2* | chr9 | c.1849G>T | p.V617F | NM_004972 |
| D-2968 | *KDM6A* | chrX | c.619+1G>A |  | NM_021140 |
| D-2983 | *KIT* | chr4 | c.2446G>T | p.D816Y | NM_000222 |
| D-2971 | *KIT* | chr4 | c.2447A>T | p.D816V | NM_000222 |
| D-2988 | *KMT2B* | chr19 | c.1378G>C | p.V460L | NM_014727 |
| D-2969 | *KMT2B* | chr19 | c.1885C>T | p.P629S | NM_014727 |
| D-2839 | *KMT2B* | chr19 | c.5501G>A | p.R1834H | NM_014727 |
| D-2071 | *KMT2B* | chr19 | c.5741G>A | p.R1914H | NM_014727 |
| D-2995 | *KMT2B* | chr19 | c.6488T>A | p.F2163Y | NM_014727 |
| D-2850 | *KMT2B* | chr19 | c.7111G>A | p.D2371N | NM_014727 |
| D-2827 | *KRAS* | chr12 | c.38G>A | p.G13D | NM_004985 |
| D-2853 | *KRAS* | chr12 | c.38G>A | p.G13D | NM_004985 |
| D-2982 | *KRAS* | chr12 | c.38G>A | p.G13D | NM_004985 |
| D-2990 | *LASP1* | chr17 | c.298G>A | p.V100I | NM_006148 |
| D-2966 | *LONP1* | chr19 | c.2035C>T | p.R679C | NM_004793 |
| D-2830 | *LONP1* | chr19 | c.1738A>G | p.T580A | NM_004793 |
| D-2978 | *LYL1* | chr19 | c.436C>A | p.P146T | NM_005583 |
| D-3006 | *MAF* | chr16 | c.1178A>C | p.Q393P | NM_005360 |
| D-2961 | *MBD1* | chr18 | c.1430G>C | p.S477T | NM_001204141 |
| D-2857 | *MBD1* | chr18 | c.853G>A | p.V285M | NM_001204137 |
| D-2849 | *MBD1* | chr18 | c.586C>T | p.R196C | NM_001204141 |
| D-2861 | *MLLT11* | chr1 | c.133G>A | p.V45I | NM_006818 |
| D-2986 | *MMACHC* | chr1 | c.641G>A | p.R214H | NM_015506 |
| D-3128 | *MSH4* | chr1 | c.397A>C | p.K133Q | NM_002440 |
| D-2863 | *MSH4* | chr1 | c.2401G>A | p.D801N | NM_002440 |
| D-3006 | *MYC* | chr8 | c.221C>G | p.P74R | NM_002467 |
| D-2982 | *MYC* | chr8 | c.992G>C | p.R331P | NM_002467 |
| D-2981 | *NCKIPSD* | chr3 | c.487_490del | p.I163fs | NM_016453 |
| D-2976 | *NF1* | chr17 | c.6802G>A | p.V2268I | NM_000267 |
| D-2851 | *NFE2* | chr12 | c. 661dupG | p.E221fs | NM_001136023 |
| D-2974 | *NFE2* | chr12 | c.661dupG | p.E221fs | NM_001136023 |
| D-2861 | *NOTCH3* | chr19 | c.6665C>T | p.P2222L | NM_000435 |
| D-2853 | *NOTCH3* | chr19 | c. 1715C>T | p.P572L | NM_000435 |
| D-2073 | *NOTCH3* | chr19 | c.6334G>A | p.G2112S | NM_000435 |
| D-2081 | *NPM1* | chr5 | c.772_773insTCTG | p.L258fs | NM_199185 |
| D-2087 | *NPM1* | chr5 | c.772_773insTCTG | p.L258fs | NM_199185 |
| D-2825 | *NPM1* | chr5 | c.772_773insTCTG | p.L258fs | NM_199185 |
| D-2842 | *NPM1* | chr5 | c.772_773insTCTG | p.L258fs | NM_199185 |
| D-2843 | *NPM1* | chr5 | c.772_773insTCTG | p.L258fs | NM_199185 |
| D-2848 | *NPM1* | chr5 | c.772_773insTCTG | p.L258fs | NM_199185 |
| D-2854 | *NPM1* | chr5 | c.772_773insTCTG | p.L258fs | NM_199185 |
| D-2856 | *NPM1* | chr5 | c.772_773insTCTG | p.L258fs | NM_199185 |
| D-2857 | *NPM1* | chr5 | c.772_773insTCTG | p.L258fs | NM_199185 |
| D-2862 | *NPM1* | chr5 | c.772_773insTCTG | p.L258fs | NM_199185 |
| D-2954 | *NPM1* | chr5 | c.772_773insTCTG | p.L258fs | NM_199185 |
| D-2959 | *NPM1* | chr5 | c.772_773insTCTG | p.L258fs | NM_199185 |
| D-2964 | *NPM1* | chr5 | c.772_773insTCTG | p.L258fs | NM_199185 |
| D-2976 | *NPM1* | chr5 | c.772_773insTCTG | p.L258fs | NM_199185 |
| D-2978 | *NPM1* | chr5 | c.772_773insTCTG | p.L258fs | NM_199185 |
| D-2985 | *NPM1* | chr5 | c.772_773insTCTG | p.L258fs | NM_199185 |
| D-2994 | *NPM1* | chr5 | c.772_773insTCTG | p.L258fs | NM_199185 |
| D-2997 | *NPM1* | chr5 | c.772_773insTCTG | p.L258fs | NM_199185 |
| D-3128 | *NPM1* | chr5 | c.772_773insTCTG | p.L258fs | NM_199185 |
| D-3009 | *NPM1* | chr5 | c.774_775insTGCA | p.L258fs | NM_199185 |
| D-2842 | *NR4A3* | chr9 | c.508G>C | p.G170R | NM_006981 |
| D-2085 | *NRAS* | chr1 | c.182A>G | p.Q61R | NM_002524 |
| D-2086 | *NRAS* | chr1 | c.182A>G | p.Q61R | NM_002524 |
| D-2841 | *NRAS* | chr1 | c.182A>G | p.Q61R | NM_002524 |
| D-2977 | *NRAS* | chr1 | c.182A>G | p.Q61R | NM_002524 |
| D-2960 | *NRAS* | chr1 | c.181C>A | p.Q61K | NM_002524 |
| D-2987 | *NRAS* | chr1 | c.181C>A | p.Q61K | NM_002524 |
| D-2967 | *NRAS* | chr1 | c.38G>A | p.G13D | NM_002524 |
| D-2859 | *NRAS* | chr1 | c.37G>C | p.G13R | NM_002524 |
| D-2077 | *NRAS* | chr1 | c.35G>A | p.G12D | NM_002524 |
| D-2078 | *NRAS* | chr1 | c.35G>A | p.G12D | NM_002524 |
| D-2988 | *NRAS* | chr1 | c.35G>A | p.G12D | NM_002524 |
| D-2071 | *NRAS* | chr1 | c.34G>T | p.G12C | NM_002524 |
| D-2085 | *NRG3* | chr10 | c.418C>A | p.P140T | NM_001010848 |
| D-2954 | *NTRK1* | chr1 | c.295G>A | p.V99M | NM_002529 |
| D-2991 | *NTRK1* | chr1 | c.652G>A | p.E218K | NM_002529 |
| D-2083 | *NUTM1* | chr15 | c.442G>A | p.V148I | NM_175741 |
| D-2863 | *NUTM1* | chr15 | c.628G>A | p.V210I | NM_175741 |
| D-2828 | *NUTM1* | chr15 | c.1370A>G | p.Q457R | NM_175741 |
| D-2978 | *PAX7* | chr1 | c.434G>A | p.R145Q | NM_013945 |
| D-2821 | *PBX1* | chr1 | c.519C>G | p.N173K | NM_002585 |
| D-2826 | *PCSK7* | chr11 | c.314G>A | p.R105K | NM_004716 |
| D-2991 | *PHF6* | chrX | c.751C>T | p.Q251X | NM_032458 |
| D-2976 | *PICALM* | chr11 | c.64G>C | p.V22L | NM_007166 |
| D-2956 | *PRAME* | chr22 | c.1514G>A | p.C505Y | NM_206956 |
| D-2851 | *PSMD2* | chr3 | c.526G>A | p.A176T | NM_002808 |
| D-2999 | *PTPN11* | chr12 | c.179G>T | p.G60V | NM_002834 |
| D-2856 | *PTPN11* | chr12 | c.215C>T | p.A72V | NM_002834 |
| D-2854 | *PTPN11* | chr12 | c.227A>T | p.E76V | NM_002834 |
| D-2964 | *PTPN11* | chr12 | c.1508G>C | p.G503A | NM_002834 |
| D-2979 | *RABEP1* | chr17 | c.2054G>A | p.R685H | NM_004703 |
| D-2957 | *RAD21* | chr8 | c.1388_1389del | p.463_463del | NM_006265 |
| D-2860 | *RAD21* | chr8 | c.54G>T | p.W18C | NM_006265 |
| D-2073 | *RAD21* | chr8 | c.1470+2T>C |  | NM_006265 |
| D-2841 | *RAD50* | chr5 | c.2047G>A | p.V683I | NM_005732 |
| D-2855 | *RAD50* | chr5 | c.3931G>A | p.V1311I | NM_005732 |
| D-2077 | *RNF213* | chr17 | c.2852A>G | p.H951R | NM_001256071 |
| D-2989 | *RNF213* | chr17 | c.11812G>A | p.E3938K | NM_001256071 |
| D-2838 | *RPN1* | chr3 | c.1696G>A | p.A566T | NM_002950 |
| D-2865 | *RUNX1* | chr21 | c.1415T>C | p.L472P | NM_001754 |
| D-2850 | *RUNX1* | chr21 | c.1210dupC | p.H404fs | NM_001754 |
| D-2963 | *RUNX1* | chr21 | c.1189dupC | p.Q397fs | NM_001754 |
| D-2981 | *RUNX1* | chr21 | c.1190A>G | p.Q397R | NM_001754 |
| D-2961 | *RUNX1* | chr21 | c.1132dupC | p.H378fs | NM_001754 |
| D-2954 | *RUNX1T1* | chr8 | c.946G>A | p.E316K | NM_175636 |
| D-2853 | *RUNX1T1* | chr8 | c.92G>A | p.R31H | NM_175634 |
| D-2982 | *SF3B1* | chr2 | c.2111T>C | p.I704T | NM_012433 |
| D-2071 | *SF3B2* | chr11 | c.512C>T | p.S171L | NM_006842 |
| D-2084 | *SMC3* | chr10 | c.121dupT | p.N40fs | NM_005445 |
| D-2981 | *SMC3* | chr10 | c.430-1G>A |  | NM_005445 |
| D-2995 | *SPRY4* | chr5 | c.745T>C | p.C249R | NM_030964 |
| D-2853 | *SPRY4* | chr5 | c. 643A>G | p.T215A | NM_030964 |
| D-2841 | *SRSF2* | chr17 | c.284C>A | p.P95H | NM_003016 |
| D-2861 | *SRSF2* | chr17 | c.284C>A | p.P95H | NM_003016 |
| D-2954 | *SRSF2* | chr17 | c.284C>A | p.P95H | NM_003016 |
| D-2965 | *SRSF2* | chr17 | c.284C>A | p.P95H | NM_003016 |
| D-2962 | *STAG2* | chrX | c.796_798del | p.266_266del | NM_006603 |
| D-2855 | *STAG2* | chrX | c.1570_71insTAAT | p.I524fs | NM_006603 |
| D-2851 | *STAG2* | chrX | c.2887C>T | p.Q963X | NM_006603 |
| D-2841 | *STAG2* | chrX | c.3130_3131insG | p.Y1044_R45delinsX | NM_006603 |
| D-2965 | *STAG2* | chrX | c.2185-2A>T |  | NM_006603 |
| D-3000 | *STIL* | chr1 | c. 227C>T | p. S76L | NM_003035 |
| D-2989 | *STRN* | chr2 | c.581C>T | p.T194M | NM_003162 |
| D-3120 | *TAF15* | chr17 | c.1372_1395del | p.458_465del | NM_003487 |
| D-2850 | *TAF15* | chr17 | c.1443_1444ins | p.G481delins | NM_003487 |
| D-2963 | *TET2* | chr4 | c.601delA | p.K201fs | NM_001127208 |
| D-2983 | *TET2* | chr4 | c.727C>T | p.Q243X | NM_001127208 |
| D-2956 | *TET2* | chr4 | c.822delC | p.I274fs | NM_001127208 |
| D-2956 | *TET2* | chr4 | c.3015delG | p.K1005fs | NM_001127208 |
| D-2855 | *TET2* | chr4 | c.3643G>T | p.E1215X | NM_001127208 |
| D-2985 | *TET2* | chr4 | c.3578G>A | p.C1193Y | NM_001127208 |
| D-2865 | *TET2* | chr4 | c.3730_3731del | p. L1244fs | NM_001127208 |
| D-2994 | *TET2* | chr4 | c.4132T>C | p.C1378R | NM_001127208 |
| D-2846 | *TET2* | chr4 | c.4659_4674del | p.Q1553fs | NM_001127208 |
| D-2081 | *TET2* | chr4 | c. 2224C>T | p. Q742X | NM_001127208 |
| D-2838 | *TLR9* | chr3 | c.1801C>T | p.R601W | NM_017442 |
| D-2967 | *TLX1* | chr10 | c.355A>G | p.S119G | NM_005521 |
| D-2086 | *TLX3* | chr5 | c.706G>A | p.A236T | NM_021025 |
| D-2085 | *TP53* | chr17 | c.473G>T | p.R158L | NM_001126115 |
| D-2074 | *TP53* | chr17 | c.428G>A | p.C143Y | NM_001126115 |
| D-2077 | *TP53* | chr17 | c.140A>G | p.H47R | NM_001126115 |
| D-2862 | *TP53* | chr17 | c.214C>G | p.L72V | NM_001126118 |
| D-2844 | *TP53* | chr17 | c.100G>A | p.V34M | NM_001126118 |
| D-2085 | *TPM3* | chr1 | c.124C>T | p.R42W | NM_153649 |
| D-2991 | *TTL* | chr2 | c.904A>G | p.I302V | NM_153712 |
| D-2961 | *U2AF1* | chr21 | c.470A>C | p.Q157P | NM_006758 |
| D-2989 | *U2AF1* | chr21 | c.104G>A | p.R35Q | NM_006758 |
| D-2960 | *U2AF1* | chr21 | c.101C>A | p.S34Y | NM_006758 |
| D-2850 | *U2AF1* | chr21 | c.101C>T | p.S34F | NM_006758 |
| D-2855 | *U2AF1* | chr21 | c.101C>T | p.S34F | NM_006758 |
| D-2966 | *U2AF1* | chr21 | c.101C>T | p.S34F | NM_006758 |
| D-2982 | *U2AF1* | chr21 | c.101C>T | p.S34F | NM_006758 |
| D-2077 | *WHSC1* | chr4 | c.304T>A | p.S102T | NM_133334 |
| D-2849 | *WHSC1L1* | chr8 | c.2031T>G | p.D677E | NM_023034 |
| D-2967 | *WT1* | chr11 | c.1321C>T | p.R441X | NM_000378 |
| D-2087 | *WT1* | chr11 | c.1250G>C | p.R417P | NM_000378 |
| D-2071 | *WT1* | chr11 | c.1237C>T | p.R413X | NM_000378 |
| D-3000 | *WT1* | chr11 | NA | NA |  |
| D-2083 | *WT1* | chr11 | c.1172T>A | p.L391X | NM_000378 |
| D-2844 | *WT1* | chr11 | c.1094_1095ins | p.A365fs | NM_000378 |
| D-2076 | *WT1* | chr11 | c.1089dupG | p.S364fs | NM_000378 |
| D-2085 | *WT1* | chr11 | c.1089dupG | p.S364fs | NM_000378 |
| D-2083 | *WT1* | chr11 | c. 1089delG | p. R363fs | NM_000378 |
| D-2832 | *WT1* | chr11 | c.1086_1096del | p.V362fs | NM_000378 |
| D-2968 | *WT1* | chr11 | c.1289G>T | p.G430V | NM_000378 |
| **Sample**  **ID** | **Gene** | **Chrom** | **Nucleotide**  **change** | **Start** | **End** |
| D-2081 | *FLT3-ITD* | chr13 | insert36bp | 28608241 | 28608242 |
| D-2825 | *FLT3-ITD* | chr13 | insert24bp | 28608262 | 28608263 |
| D-2826 | *FLT3-ITD* | chr13 | insert33bp | 28608252 | 28608253 |
| D-2832 | *FLT3-ITD* | chr13 | insert66bp | 28608237 | 28608238 |
| D-2843 | *FLT3-ITD* | chr13 | insert60bp | 28608216 | 28608217 |
| D-2959 | *FLT3-ITD* | chr13 | insert66bp | 28608262 | 28608263 |
| D-2968 | *FLT3-ITD* | chr13 | insert21bp | 28608255 | 28608256 |
| D-2975 | *FLT3-ITD* | chr13 | insert21bp | 28608262 | 28608263 |
| D-2978 | *FLT3-ITD* | chr13 | insert21bp | 28608261 | 28608262 |
| D-2985 | *FLT3-ITD* | chr13 | insert36bp | 28608219 | 28608220 |
| D-2995 | *FLT3-ITD* | chr13 | insert78bp | 28608288 | 28608289 |
| D-3000 | *FLT3-ITD* | chr13 | insert21bp | 28608295 | 28608296 |
| D-3128 | *FLT3-ITD* | chr13 | insert63bp | 28608276 | 28608277 |
| D-3009 | *FLT3-ITD* | chr13 | insert51bp | 28608233 | 28608234 |
