# Supplementary material for: Mutational spectrum and risk stratification of intermediate-risk acute myeloid leukemia patients based on next-generation sequencing
Source: Oncotarget. 2016 Jan 27;7(22):32065–78. doi: 10.18632/oncotarget.7028 (PMC5077997; doi:10.18632/oncotarget.7028)
Supplement: Supplementary file 4 [file oncotarget-07-32065-s004.docx]

**Table S3: Validation of gene mutations by sanger sequencing**

| Gene  name | Sample  ID | Version | Position | Geno  type | Backward  15 bases | Sanger Result | Validated |
| --- | --- | --- | --- | --- | --- | --- | --- |
| *DNMT3A* | D-3009 | Hg19 | 25457158 | G/A | caaaatactccttca | G/A | Yes(PCR/Sanger) |
| *DNMT3A* | D-2081 | Hg19 | 25457242 | C/T | ggctcatgttggaga | C/T | Yes(PCR/Sanger) |
| *DNMT3A* | D-2842 | Hg19 | 25457242 | C/T | ggctcatgttggaga | C/T | Yes(PCR/Sanger) |
| *DNMT3A* | D-2856 | Hg19 | 25457242 | C/T | ggctcatgttggaga | C/T | Yes(PCR/Sanger) |
| *DNMT3A* | D-2861 | Hg19 | 25457242 | C/T | ggctcatgttggaga | C/T | Yes(PCR/Sanger) |
| *DNMT3A* | D-2961 | Hg19 | 25457242 | C/T | ggctcatgttggaga | C/T | Yes(PCR/Sanger) |
| *DNMT3A* | D-2968 | Hg19 | 25457242 | C/T | ggctcatgttggaga | C/T | Yes(PCR/Sanger) |
| *DNMT3A* | D-2985 | Hg19 | 25457242 | C/T | ggctcatgttggaga |  | NA(sample lacking) |
| *DNMT3A* | D-3128 | Hg19 | 25457242 | C/T | ggctcatgttggaga | C/T | Yes(PCR/Sanger) |
| *DNMT3A* | D-2978 | Hg19 | 25457242 | C/G | ggctcatgttggaga | C/G | Yes(PCR/Sanger) |
| *DNMT3A* | D-2857 | Hg19 | 25457243 | G/A | gctcatgttggagac | G/A | Yes(PCR/Sanger) |
| *DNMT3A* | D-2959 | Hg19 | 25457243 | G/A | gctcatgttggagac | G/A | Yes(PCR/Sanger) |
| *DNMT3A* | D-2987 | Hg19 | 25458692 | G/T | aactagatgaagagg | G/T | Yes(PCR/Sanger) |
| *DNMT3A* | D-2964 | Hg19 | 25463293 | A/T | ctcaaagaagagcc | A/T | Yes(PCR/Sanger) |
| *IDH2* | D-2957 | Hg19 | 90631838 | C/T | tgccaatggtgatgg | C/T | Yes(PCR/Sanger) |
| *IDH2* | D-2842 | Hg19 | 90631934 | C/T | ggatagttccattgg | C/T | Yes(PCR/Sanger) |
| *IDH2* | D-2848 | Hg19 | 90631934 | C/T | ggatagttccattgg | C/T | Yes(PCR/Sanger) |
| *IDH2* | D-2861 | Hg19 | 90631934 | C/T | ggatagttccattgg | C/T | Yes(PCR/Sanger) |
| *IDH2* | D-2862 | Hg19 | 90631934 | C/T | ggatagttccattgg | C/T | Yes(PCR/Sanger) |
| *IDH2* | D-2954 | Hg19 | 90631934 | C/T | ggatagttccattgg | C/T | Yes(PCR/Sanger) |
| *IDH2* | D-2961 | Hg19 | 90631934 | C/T | ggatagttccattgg | C/T | Yes(PCR/Sanger) |
| *IDH2* | D-2968 | Hg19 | 90631934 | C/T | ggatagttccattgg | C/T | Yes(PCR/Sanger) |
| *IDH2* | D-2981 | Hg19 | 90631934 | C/T | ggatagttccattgg | C/T | Yes(PCR/Sanger) |
| *IDH2* | D-2989 | Hg19 | 90631934 | C/T | ggatagttccattgg | C/T | Yes(PCR/Sanger) |
| *IDH2* | D-2996 | Hg19 | 90631934 | C/T | ggatagttccattgg | C/T | Yes(PCR/Sanger) |
| *NRAS* | D-2960 | Hg19 | 115256530 | G/T | tccagctgtatccag | G/T | Yes(PCR/Sanger) |
| *NRAS* | D-2987 | Hg19 | 115256530 | G/T | tccagctgtatccag | G/T | Yes(PCR/Sanger) |
| *NRAS* | D-2967 | Hg19 | 115258744 | C/T | cacctgctccaacca | C/T | Yes(PCR/Sanger) |
| *NRAS* | D-2859 | Hg19 | 115258745 | C/G | acctgctccaaccac | C/G | Yes(PCR/Sanger) |
| *NRAS* | D-2071 | Hg19 | 115258748 | C/A | tgctccaaccaccac | C/A | Yes(PCR/Sanger) |
| *TET2* | D-2983 | Hg19 | 106155826 | C/T | agaaaaccacatctc | C/T | Yes(PCR/Sanger) |
| *TET2* | D-2081 | Hg19 | 106157323 | C/T | agcaacagcagcaa | C/T | Yes(PCR/Sanger) |
| *TET2* | D-2985 | Hg19 | 106164068 | G/A | tcctattgctaagtg |  | NA(sample lacking) |
| *TET2* | D-2855 | Hg19 | 106164775 | G/T | agcgagctggccac | G/T | Yes(PCR/Sanger) |
| *TET2* | D-2994 | Hg19 | 106190854 | T/C | gtgctcatgcccaca | T/C | Yes(PCR/Sanger) |
| *WT1* | D-2967 | Hg19 | 32413578 | G/A | ctgacaagttttaca | G/A | Yes(PCR/Sanger) |
| *WT1* | D-2968 | Hg19 | 32413610 | C/A | ctaaatggacagaga | C/A | Yes(PCR/Sanger) |
| *WT1* | D-2087 | Hg19 | 32414250 | C/G | gagaaaaccttcgtt | C/G | Yes(PCR/Sanger) |
| *WT1* | D-2071 | Hg19 | 32414263 | G/A | ttcacagtccttgaa | G/A | Yes(PCR/Sanger) |
| *RNF213* | D-2989 | Hg19 | 78338294 | G/A | agagccgcgtcccc | G/A | Yes(PCR/Sanger) |
