# Supplementary material for: Mutational spectrum and risk stratification of intermediate-risk acute myeloid leukemia patients based on next-generation sequencing
Source: Oncotarget. 2016 Jan 27;7(22):32065–78. doi: 10.18632/oncotarget.7028 (PMC5077997; doi:10.18632/oncotarget.7028)
Supplement: Supplementary file 5 [file oncotarget-07-32065-s005.docx]

**Table S4: The clinical features of** **single mutational analysis**

**(1) Relationships between mutations and age, WBC count**

| **V****ariable** | **Numbers of cases** | | ***P* value** |
| --- | --- | --- | --- |
|  | ≥50y | <50y |  |
| *DNMT3A* mut | 10 | 6 | 0.044 |
| *DNMT3A* wt | 28 | 51 |  |

| **V****ariable** | **Numbers of cases** | | ***P* value** |
| --- | --- | --- | --- |
|  | ≥40y | <40y |  |
| *NPM1* mut | 17 | 3 | 0.034 |
| *NPM1*  wt | 42 | 33 |  |

| **V****ariable** | **WBC count at diagnosis** | ***P* value** |
| --- | --- | --- |
| *FLT3-ITD* mut | 129.21 (7.5-405.13) | 0.001 |
| *FLT3-ITD* wt | 15.235 (0.87-186.6) |  |

| **Variable** | **WBC count at diagnosis** | ***P* value** |
| --- | --- | --- |
| *IDH2* mut | 5.3 (0.9-49.4) | 0.032 |
| *IDH2* wt | 24.19 (0.87-405.13) |  |

Mutation frequencies were compared using Pearson’s χ^2^ test. Median and range of WBC counts are shown. *FLT3-ITD* mutations were significantly associated with high leukocyte counts and *IDH2* mutations were associated with lower leukocyte counts. Differences were evaluated using a Mann-Whitney U-test.

**(2) Relationships between mutations and immunophenotype**

| **V****ariable** | **Numbers of cases** | | ***P* value** |
| --- | --- | --- | --- |
|  | CD117(+) | CD117(-) |  |
| *DNMT3A* mut | 10 | 6 | 0.038 |
| *DNMT3A* wild | 67 | 12 |  |

| **Vari****able** | **Numbers of cases** | | ***P* value** |
| --- | --- | --- | --- |
|  | CD7(+) | CD7(-) |  |
| *CEBPA* mut | 13 | 14 | 0.042 |
| *CEBPA* wild | 18 | 50 |  |

| **Muta****tions** | **Numbers of mutated cases** | | ***P* value** |
| --- | --- | --- | --- |
|  | CD34(+) | CD34(-) |  |
| *NPM1* | 7 | 11 | 0.000  0.028  0.000 |
| *DNMT3A* | 8 | 7 |  |
| *CEBPA* | 26 | 1 |  |

| **Muta****tions** | **Numbers of mutated cases** | | ***P* value** |
| --- | --- | --- | --- |
|  | CD33(+) | CD33(-) |  |
| *NPM1* | 20 | 0 | 0.000  0.027 |
| *DNMT3A* | 12 | 4 |  |

| **Mutations** | **Numbers of mutated cases** | | ***P* value** |
| --- | --- | --- | --- |
|  | HLA-DR(+) | HLA-DR(-) |  |
| *NPM1* | 11 | 9 | 0.007  0.037 |
| *IDH2* | 6 | 5 |  |

*NMP1* mutations were associated with lower CD34- and HLA-DR-positive rates while higher CD33-positive rates; *DNMT3A* mutations were associated with lower CD34-, CD33- and CD117-positive rates; *CEBPA* mutations were associated with higher CD34-and CD7- positive rates; and *IDH2* mutations were associated with lower HLA-DR-positive rates. *P* values were evaluated by Fisher’s exact test or Pearson’s χ^2^ test.
